# MpaR-driven expression of an orphan terminal oxidase subunit supports *Pseudomonas aeruginosa* biofilm respiration and development during cyanogenesis

**DOI:** 10.1128/mbio.02926-23

**Published:** 2023-12-19

**Authors:** Marina K. Smiley, Doran C. Sekaran, Farhad Forouhar, Erica Wolin, Marko Jovanovic, Alexa Price-Whelan, Lars E. P. Dietrich

**Affiliations:** 1 Department of Biological Sciences, Columbia University, New York, New York, USA; 2 Herbert Irving Comprehensive Cancer Center, Columbia University, New York, New York, USA; University of Washington School of Medicine, Seattle, Washington, USA

**Keywords:** cyanide, biofilms, *Pseudomonas aeruginosa*, transcriptional regulation, terminal oxidase

## Abstract

*Pseudomonas aeruginosa* is a common, biofilm-forming pathogen that exhibits complex pathways of redox metabolism. It produces four different types of terminal oxidases for aerobic respiration, and for one of these, the *cbb*_3_-type terminal oxidases, it has the capacity to produce at least 16 isoforms encoded by partially redundant operons. It also produces small-molecule virulence factors that interact with the respiratory chain, including the poison cyanide. Previous studies had indicated a role for cyanide in activating expression of an “orphan” terminal oxidase subunit gene called *ccoN4* and that the product contributes to *P. aeruginosa* cyanide resistance, fitness in biofilms, and virulence, but the mechanisms underlying this process had not been elucidated. Here, we show that the regulatory protein MpaR, which is predicted to be a pyridoxal phosphate-binding transcription factor and is encoded just upstream of *ccoN4*, controls *ccoN4* expression in response to endogenous cyanide. Paradoxically, we find that cyanide production is required to support CcoN4’s contribution to respiration in biofilms. We identify a palindromic motif required for cyanide- and MpaR-dependent expression of *ccoN4* and co-expressed adjacent loci. We also characterize the regulatory logic of this region of the chromosome. Finally, we identify residues in the putative cofactor-binding pocket of MpaR, which are required for *ccoN4* expression. Together, our findings illustrate a novel scenario in which the respiratory toxin cyanide acts as a signal to control gene expression in a bacterium that produces the compound endogenously.

**IMPORTANCE:**

Cyanide is an inhibitor of heme-copper oxidases, which are required for aerobic respiration in all eukaryotes and many prokaryotes. This fast-acting poison can arise from diverse sources, but mechanisms by which bacteria sense it are poorly understood. We investigated the regulatory response to cyanide in the pathogenic bacterium *Pseudomonas aeruginosa*, which produces cyanide as a virulence factor. Although *P. aeruginosa* has the capacity to produce a cyanide-resistant oxidase, it relies primarily on heme-copper oxidases and even makes additional heme-copper oxidase proteins specifically under cyanide-producing conditions. We found that the protein MpaR controls expression of cyanide-inducible genes in *P. aeruginosa* and elucidated the molecular details of this regulation. MpaR contains a DNA-binding domain and a domain predicted to bind pyridoxal phosphate (vitamin B6), a compound that is known to react spontaneously with cyanide. These observations provide insight into the understudied phenomenon of cyanide-dependent regulation of gene expression in bacteria.

## INTRODUCTION

Biofilms are crowded structures containing cells and secreted polymers that form an adherent matrix. As a biofilm grows, internal chemical gradients become established because (i) cells near the biofilm edge consume substrates such as O_2_ before these resources can travel through the structure and (ii) cells in separate biofilm subregions release distinct products. Cells in microniches within biofilms use different tactics to survive, thus decreasing the efficacy of single-target treatments (1–5).

The opportunistic pathogen *Pseudomonas aeruginosa* is known for its respiratory versatility and its production of small molecules that interact with the respiratory chain (6–9). Depending on the growth conditions, it can synthesize four major types of terminal oxidases, i.e., enzyme complexes that catalyze the final step of aerobic respiration, including high-affinity *cbb*_3_-type oxidases (“Ccos”). The production of small molecules that interact with the respiratory chain is generally controlled by quorum-sensing pathways and/or regulators that respond to low-oxygen conditions (7, 10, 11). Among these compounds are the *P. aeruginosa* phenazines, a class of redox-cycling compounds that have been implicated in anaerobic survival (12, 13) and in electron transfer by the respiratory chain (9, 14). Together, the terminal oxidases and phenazines provide extensive and varied potential routes of metabolic electron flow for *P. aeruginosa* cells in biofilms. Adding to this complexity is the fact that *P. aeruginosa* has the capacity to produce cyanide, a potent inhibitor of heme-copper oxidases including *cbb*_3_-type oxidases (15–18). These observations raise questions about how *P. aeruginosa* mediates electron flow when O_2_ limitation and cyanide production threaten respiratory function.

**Fig 1:**
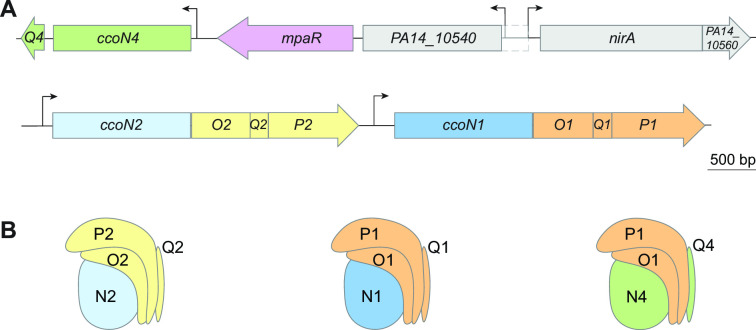
Genomic arrangement of *cco* genes. (**A**) Arrangement of *mpaR* and *cco* genes on the *P. aeruginosa* PA14 chromosome. The locations of transcription start sites were determined using data in reference (23). The depicted size of the *PA14_10540* open reading frame (ORF) is based on the transcription start site indicated by RNAseq data; a larger ORF (area with dashed outline) is predicted by The Pseudomonas Genome Database and BioCyc. (**B**) Cartoon representations of assembled *cbb*_3_-type terminal oxidase complexes, based on the Cco structure from *P. stutzeri* (PDB: 3mk7) (24) and including a hybrid isoform that combines Cco subunits encoded by different operons.

The *P. aeruginosa* genome contains multiple, differentially regulated genetic loci that produce full or partial Cco complexes, with functionally interchangeable subunits (19) (Fig. 1A and B). One of genes, *ccoN4*, encodes a so-called “orphan” Cco subunit that contributes to wild-type (WT) biofilm formation and competitive fitness in biofilms (14). *ccoN4* is part of a cluster that is strongly induced by cyanide (20). At first, it seems odd that cyanide induces *ccoN4* expression because cyanide is known for its specific inhibition of oxidases like the Ccos (21). Nevertheless, studies have shown that *ccoN4* contributes to resistance when exogenous cyanide is added to liquid cultures (19, 20). Although *P. aeruginosa* can produce a cyanide-insensitive oxidase (CIO) that does not contain a heme-copper center, a drawback to the use of this oxidase is that it has a low affinity for O_2_ relative to the Cco complexes. Moreover, viability studies have shown that CIO is not the sole contributor to *P. aeruginosa* cyanide resistance (19, 22). These observations suggest that the CcoN4 subunit is functional in the presence of cyanide and that its use may be preferred when high O_2_ affinity is beneficial such as in hypoxic biofilm settings.

*ccoN4* lies adjacent to *mpaR* (*PA14_10530*), which codes for a GntR family transcription factor. In this study, we investigated the role of MpaR in *ccoN4* and *mpaR* expressions and its responsiveness to cyanide. Our findings support a model for the role of this regulatory protein in cyanide resistance across depth in *P. aeruginosa* biofilms.

## RESULTS

### CcoN4 contributes to tetrazolium chloride (TTC) reduction in a cyanide-dependent manner

Among the various types of terminal oxidases produced by *P. aeruginosa*, the *cbb*_3_ type is most important for growth under standard laboratory conditions and appears to be the only one that is produced constitutively (25–28). A minimal functional *cbb*_3_-type complex comprises two subunits, CcoN and CcoO; two additional and nonessential subunits, CcoP and CcoQ, have been implicated in assembly or electron transfer (29–31). Although *cco* genes (encoding *cbb*_3_-type oxidase subunits) are found in diverse bacteria, the *P. aeruginosa* genome is unusual in that it contains two complete and two partial *cco* operons (14, 19). The *cco1* and *cco2* operons lie adjacent to each other and are each sufficient to produce a full Cco complex (Fig. 1A and B); the *ccoN3Q3* and *ccoN4Q4* operons are found at independent locations in the genome and code for orphan subunits that can replace their corresponding subunits in Cco1 or Cco2 to produce hybrid complexes. A study conducted in *P. aeruginosa* PAO1 tested 16 possible isoforms of Cco, containing subunits encoded by different operons in distinct combinations, and demonstrated that they were all sufficient to support aerobic growth similar to that seen for the parent strain (19). However, several lines of evidence point to unique physiological roles for the CcoN4 subunit. Our prior work in *P. aeruginosa* PA14 has implicated *ccoN4* in (i) phenazine reduction and fitness during biofilm growth and (ii) virulence in a *C. elegans* slow-kill assay (14). In addition, work by others has indicated that *ccoN4* contributes specifically to resistance to exogenous cyanide (19, 20). These observations motivated us to test the contribution of CcoN4 to respiration in the presence of endogenously produced cyanide.

**Fig 2:**
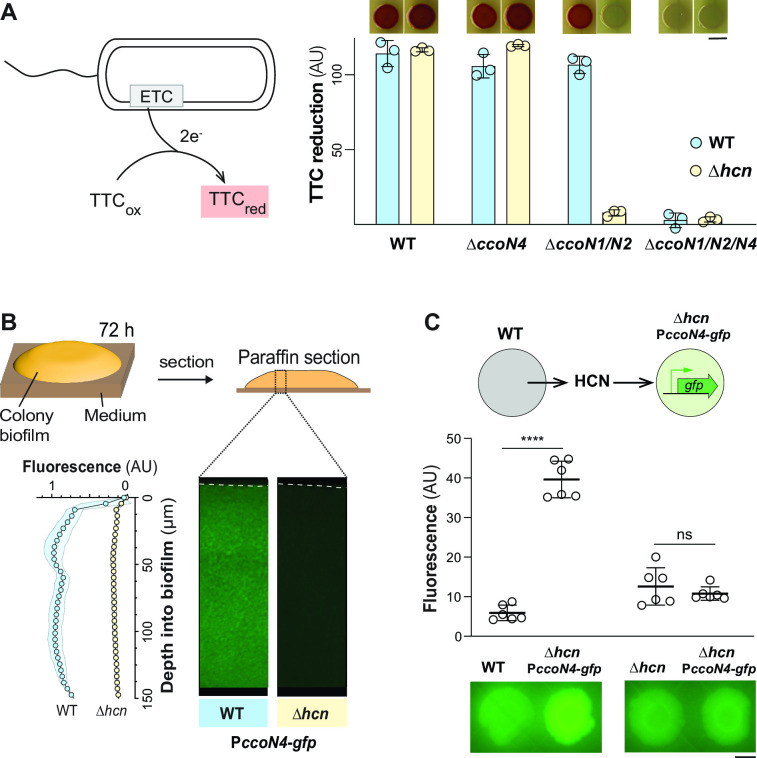
*ccoN4* contributes to respiration and is induced by endogenous cyanide. (**A**) Left: TTC reduction by the electron transport chain (ETC) produces a red compound. Right: TTC reduction by colony biofilms of the indicated strains, quantified as the average saturation in the red hue for all pixels in the colony area. Data points represent biological replicates, and error bars indicate standard deviation. Scale bar is 5 mm. (**B**) Top: colony biofilms were grown for 3 days before harvesting and preparation of thin sections. Bottom left: activity of the P*ccoN4-gfp* reporter construct across depth in colony biofilms. Bottom right: representative thin-section images of the indicated strains, each containing the P*ccoN4-gfp* reporter construct. Dotted lines indicate the top of each biofilm. Experiments were performed with biological triplicates, and shading indicates standard deviation. (**C**) Top: assay for HCN-dependent induction at a distance. Middle: top-down quantification of fluorescence for adjacent biofilms grown in the HCN-dependent induction assay. Error bars represent the standard deviation of biological replicates. *P* values were calculated using unpaired, two tailed *t* tests (**** *P* < 0.0001). Biofilms were grown for 3 days on 1% tryptone 1% agar before scanning. Scale bar is 5 mm.

To ask whether endogenous cyanide affects respiration via specific CcoN subunits, we used a TTC-based assay in which the colorless dye changes to red when reduced by cytochrome c oxidase-dependent respiration (32) (Fig. 2A). We examined TTC reduction in *P. aeruginosa* PA14 biofilms of single and combinatorial mutants lacking *ccoN* genes and/or the *hcnABC* operon (“*hcn*”), the latter of which is required for cyanide synthesis. We found that CcoN4 contributes to dye reduction in this assay, particularly in the ∆*ccoN1/N2* background, as described previously (14). We also found that this contribution is dependent on the presence of *hcnABC* (Fig. 2A). This result is consistent with the prior observation, made in *P. aeruginosa* strain PAO1, that endogenous cyanide induces *ccoN4Q4* expression (20). Paradoxically, the role of cyanide in *ccoN4* induction appears to create a scenario where a respiratory toxin is necessary for substantial respiration in the ∆*ccoN1/N2* background.

### Endogenous cyanide boosts transcription of *ccoN4* in *P. aeruginosa* PA14 biofilms

Cyanide-dependent control of *ccoN4Q4* expression has previously been characterized in *P. aeruginosa* PAO1 liquid cultures (19, 20). However, cyanide synthesis is regulated in response to cell density, and studies comparing PAO1 and PA14 have described strain-dependent differences in quorum-sensing controlled processes (33, 34). Furthermore, the condition dependence of cyanide production (10, 35) could affect its regulation in chemically heterogeneous biofilm subzones (10, 35). We, therefore, sought to directly examine the effect of endogenously produced cyanide on activity of the *ccoN4* promoter in PA14 liquid cultures and biofilms. We created a reporter construct in which *ccoN4*’s putative promoter region drives expression of *gfp* (P*ccoN4-gfp*) and moved it into a neutral chromosomal site in the WT and a ∆*hcn* mutant (36). Planktonic fluorescence assays indicate that *ccoN4* transcription is induced at the end of the exponential growth phase and that it is not induced in the ∆*hcn* background (Fig. S1). This result is in line with measurements of P*ccoN4-lacZ* reporter activity made by Hirai et al. (19).

To examine the role of cyanide in *ccoN4* expression in biofilms, we grew macrocolony biofilms for 3 days on 1% tryptone 1% agar medium, embedded them in paraffin, and prepared thin sections for microscopic imaging. We focused our analysis on the top 150 µm of the biofilm, which includes the transition from oxic to anoxic subzones (14), because the syntheses of cyanide and some terminal oxidases are O_2_ regulated (10, 26, 28). In the WT background, the *ccoN4* transcriptional reporter showed high levels of expression across most of the biofilm depth, with a reproducible peak in expression at a depth of 40–50 µm (Fig. 2B). *ccoN4* expression across depth was greatly reduced in the cyanide-null background. Finally, because cyanide is a volatile compound, cyanide-producing strains should be able to chemically complement cyanide-null mutants when they are grown in proximity. To test this, we grew WT and ∆*hcn* biofilms near, but not touching, ∆*hcn* P*ccoN4-gfp* biofilms on an agar plate. We found that incubation of cyanide-null biofilms near WT (cyanide producing) biofilms was sufficient to activate *ccoN4* expression (Fig. 2C). In sum, PA14 planktonic cultures and biofilms both show induction of *ccoN4* expression by endogenous cyanide, with biofilms showing small but reproducible variations in expression across depth.

### MpaR is required for *ccoN4* expression

In *P. aeruginosa* strain PAO1, high induction by cyanide was observed not just for the *ccoN4Q4* operon (ORFS PA4133-4134) but also the four genes upstream, which are ORFS PA4129-PA4132 (20). The chromosomal arrangement of the six corresponding genes in strain PA14 is shown in Fig. 1A. Transcriptional analyses indicate that the gene pairs *nirA-PA14_10560* and *PA14_10540-mpaR* are each co-transcribed (23). *nirA* codes for a nitrite reductase (37), and *PA14_10540* codes for an ortholog of CcoG, a cupric reductase involved in the assembly of *cbb*_3_-type oxidases (38). We were intrigued by the presence of *mpaR*, coding for a GntR-family transcriptional regulator, directly adjacent to *ccoN4* because transcription factors are often encoded upstream of (one of) their targets. A recent RNAseq analysis using a *P. aeruginosa* PAO1 ∆*mpaR* mutant and a twofold cutoff showed that MpaR affects the expression of 275 genes and highlighted its integration into *P. aeruginosa*’s quorum-sensing network (Fig. S2) (39). The transcripts for ORFs PA4129-PA4134 (which includes *ccoN4Q4*) were among those that showed the largest decreases in abundance in ∆*mpaR* when compared to the WT. To directly test whether MpaR is required for *ccoN4* expression in strain PA14, we created a markerless *mpaR* deletion (∆*mpaR*) and inserted the P*ccoN4-gfp* reporter construct at a neutral chromosomal site. To test whether *mpaR* affects the expression of other *cco* genes, we also deleted *mpaR* in strains containing *gfp* reporter constructs for P*ccoN1* and P*ccoN2*. Finally, to test whether Anr, an O_2_-sensitive transcription factor required for *cco2* expression (28), is important for P*ccoN4* activity, we moved the P*ccoN4-gfp* construct into the ∆*anr* background. We used these reporter strains to assess promoter activity during growth in liquid cultures and across depth in biofilms.

**Fig 3:**
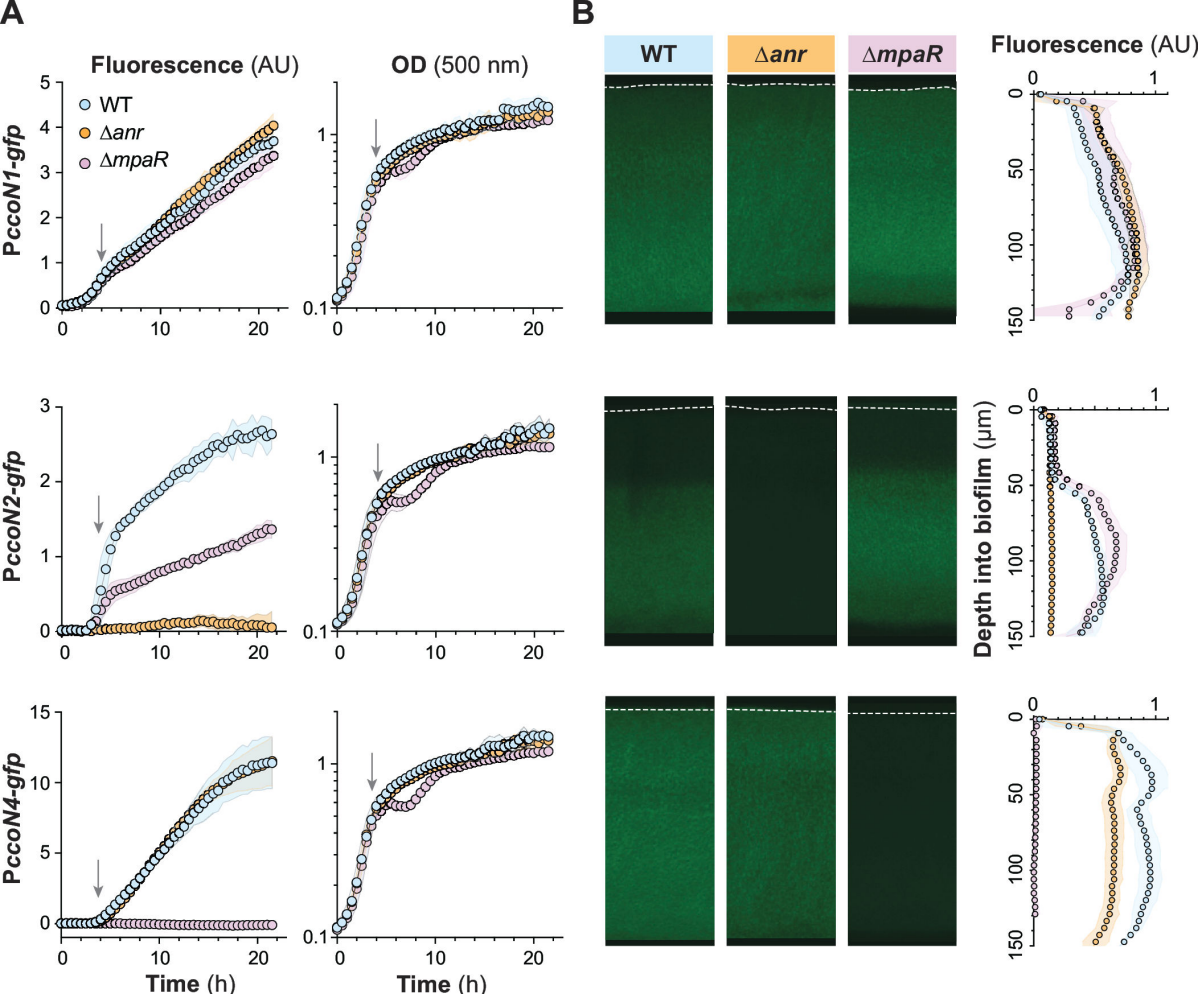
*P. aeruginosa* Anr and MpaR control *cco* gene expression in liquid cultures and biofilms. (**A**) Activities of the *ccoN1*, *ccoN2*, and *ccoN4* promoters during growth in liquid culture (1% tryptone) reported as GFP fluorescence. Data represent the mean of six biological replicates with shading indicating standard deviation. Arrow represents the onset of stationary phase. (**B**) Thin sections of biofilms grown for 3 days. The quantification on the right shows the mean of biological triplicates, and shading indicates standard deviation.

We found that planktonic growth of the ∆*mpaR* strain is altered, relative to the WT, at the transition from exponential to stationary phase (Fig. 3A). In addition to genes that enhance cyanide resistance, the MpaR regulon includes genes that code for (i) primary metabolic and respiratory enzymes, (ii) proteins that contribute to cell density-dependent processes (i.e., quorum sensing), and (iii) sigma factors, all of which may influence exponential-to-stationary phase growth kinetics. Examining reporter fluorescence in liquid cultures, we further found that MpaR is required for expression of *ccoN4* while its loss has minimal effect on *cco1* expression and only a partial effect on the expression of *cco2*, which is a well-established target of Anr (26, 28, 40) (Fig. 3A). Complementing the *mpaR* deletion restored *ccoN4* expression to WT levels (Fig. S3). These observations are consistent with RNAseq data from reference (39), in which the *ccoN4* and *ccoP2* transcripts were ~98-fold and ~2-fold, respectively, less abundant in a ∆*mpaR* mutant compared to its parent strain, *P. aeruginosa* PAO1 (39). Interestingly, in contrast to liquid culture, the loss of *mpaR* showed little to no effect on *cco2* expression in biofilms (Fig. 3). Additionally, while we observed that deletion of *anr* does not affect *ccoN4* expression in planktonic growth, it has a small downregulatory effect during biofilm growth which may arise from the fact that Anr promotes cyanogenesis (10). In both modes of growth, reporter data indicated that neither Anr nor MpaR contribute to *cco1* expression, which is in line with prior studies (26). Finally, having observed that *ccoN4* contributes to respiration in biofilms and that MpaR regulates this gene, we tested whether respiratory activity was altered in ∆*mpaR* biofilms relative to those formed by the WT. We found that *mpaR* was required for WT levels of TTC reduction specifically in the cyanide-producing background, consistent with its role in inducing *ccoN4* (Fig. S4).

### Identification of a palindromic motif required for *ccoN4* expression

**Fig 4:**
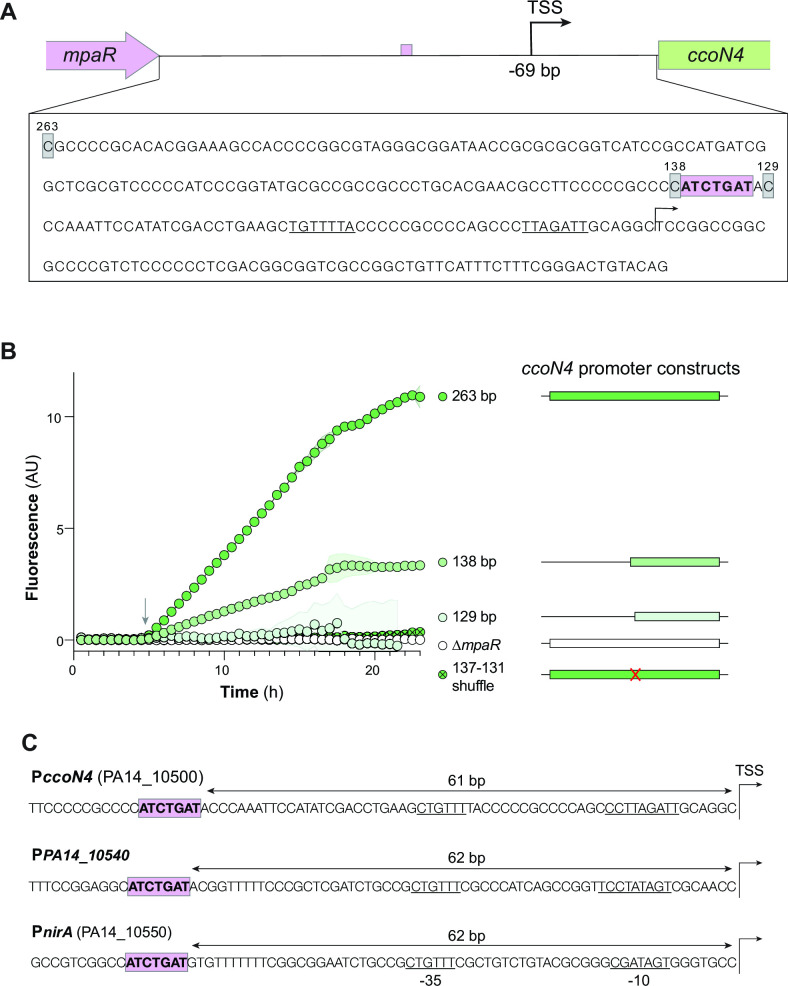
Mutational analysis of the *ccoN4* promoter region reveals a putative MpaR binding site. (**A**) Top: schematic showing genomic orientation and transcription start site of the *mpaR-ccoN4* intergenic region, created using data provided by Wurtzel et al. (23). The putative MpaR binding site identified in this study is indicated by a purple rectangle. TSS, transcriptional start site. Bottom: sequence of the *mpaR-ccoN4* intergenic region. Numbers indicate the nucleotide count, running backwards from the ATG. Putative −35 and −10 sites are underlined. (**B**) Expression dynamics of each of the indicated reporter strains during growth in liquid culture. The schematics shown on the right represent the portion of the *mpaR-ccoN4* intergenic region included in the reporter construct, with “263 bp” constituting the full intergenic region. A strain containing the full intergenic region in the ∆*mpaR* background is shown as a negative control, and the “137–131 shuffle” strain contains the full intergenic region with bp 137–131 changed to GATATCA. Values shown represent the average of three biological replicates, and shading indicates standard deviation. Arrow indicates the onset of stationary phase. (**C**) Promoter regions of the three transcription start sites in the “cyanide-inducible gene cluster” (20, 23). All three contain the same short palindromic repeat 61–62 bp upstream of the TSS (highlighted in purple). Predicted −10 and −35 sites are underlined.

We next sought to characterize features of the *ccoN4* promoter that underpin MpaR-dependent expression. According to a genome-wide analysis carried out by Wurtzel et al., the transcription start site (TSS) for *ccoN4* is 69 bp upstream of its start codon (Fig. 4A) (23). To identify regions of the *ccoN4* promoter that are required for MpaR-dependent expression, we created a series of reporter constructs in which *ccoN4* promoter sequences of varying length drove *gfp* expression. We found that shortening the promoter region from 263 to 138 bp decreased but did not abrogate *gfp* expression (Fig. 4B), indicating the involvement of additional factors that promote *ccoN4* expression [in this context, a GC-rich sequence at positions 147 to 138 bp upstream of the start codon is noteworthy (39)]. However, further shortening the promoter length from 138 to 129 bp fully prevented expression of our *gfp* reporter (Fig. 4B) indicating that the 138–129-bp sequence is required for *ccoN4* expression. Intriguingly, this stretch contains a short palindromic motif (ATCTGAT), which also appears in the promoter regions of the adjacent genes *PA14_10540* and *nirA*—the two other targets in the vicinity that may also be under the control of MpaR (Fig. 4C) (39). We shuffled these seven base pairs (ATCTGAT to GATATCA) in the full-length promoter construct and found that they are required for gene expression (Fig. 4B).

Prior work had identified a GC-rich, putative MpaR-binding motif upstream of *mvfR* (*pqsR*), which codes for a global regulator of quorum sensing (39, 41). The GC-rich sequence at 147 to 138 bp upstream of the *ccoN4* start codon, however, may contribute to but is not required for *ccoN4* expression (Fig. S5). Identification of the binding motif in the *mvfR* promoter was carried out *in vitro* and with a truncated version of MpaR (39), which could contribute to differences we observed when examining MpaR-dependent P*ccoN4* activity *in vivo*. Although the GC-rich sequence in the *ccoN4* promoter was not required for expression, we instead found that an adjacent palindromic (ATCTGAT) motif was required and, therefore, might constitute an alternate MpaR binding site.

### Constitutive *mpaR* expression reveals that cyanide is required for full MpaR function

**Fig 5:**
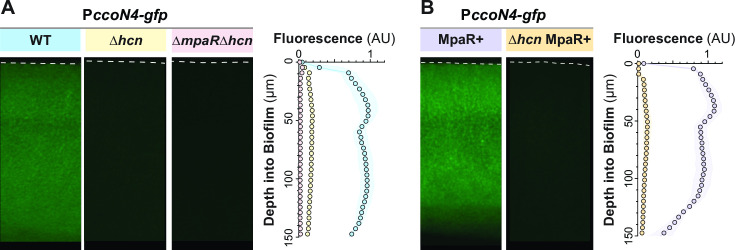
Endogenous cyanide is required for MpaR-driven expression of *ccoN4*. (**A and B**) Representative thin-section images of the indicated strains, each containing the P*ccoN4-gfp* reporter construct. Biofilms were grown for 3 days before harvesting and preparation of thin sections. Quantification is shown on the right. Dotted lines indicate the top of each biofilm. For quantification of fluorescence across biofilm depth, experiments were performed with biological triplicates, and shading indicates standard deviation.

Transcriptomic studies have reported that (i) *mpaR* and its neighboring genes are simultaneously induced by cyanide and (ii) *mpaR*’s neighboring genes are downregulated in a ∆*mpaR* background (20, 39). To further characterize the effects of MpaR and cyanide, we deleted *mpaR* in the ∆*hcn* P*ccoN4-gfp* background and found that the low level of *ccoN4* expression observed in ∆*hcn* was eliminated (Fig. 5A). The abolishment of basal *ccoN4* expression by *mpaR* deletion in ∆*hcn* hints at the possibility that MpaR has low cyanide-independent activity that is boosted by endogenous cyanide. Together, these observations also suggest two possible scenarios for the regulation of the MpaR/cyanide-controlled gene cluster. In one scenario, cyanide induces production of MpaR and MpaR, in turn, induces transcription of the rest of the gene cluster in a cyanide-independent manner. Alternatively, cyanide might activate MpaR (e.g., by stimulating a conformational change in the protein). To distinguish between these scenarios, we decoupled *mpaR* transcription from MpaR activity by cloning the *mpaR* gene after a synthetic constitutive promoter (42) and inserted this construct at the neutral *glmS* site in a ∆*mpaR* P*ccoN4-gfp* background. Biofilms formed by this MpaR+ P*ccoN4-gfp* strain show high levels of *ccoN4* expression (Fig. 5B). Removing the capacity to produce cyanide in this strain brought fluorescence down to a level similar to that observed for ∆*hcn* P*ccoN4-gfp* (Fig. 5B). These results show that cyanide is directly required for full expression of *ccoN4*.

### Autoregulation of the *PA14_10540-mpaR* operon

**Fig 6:**
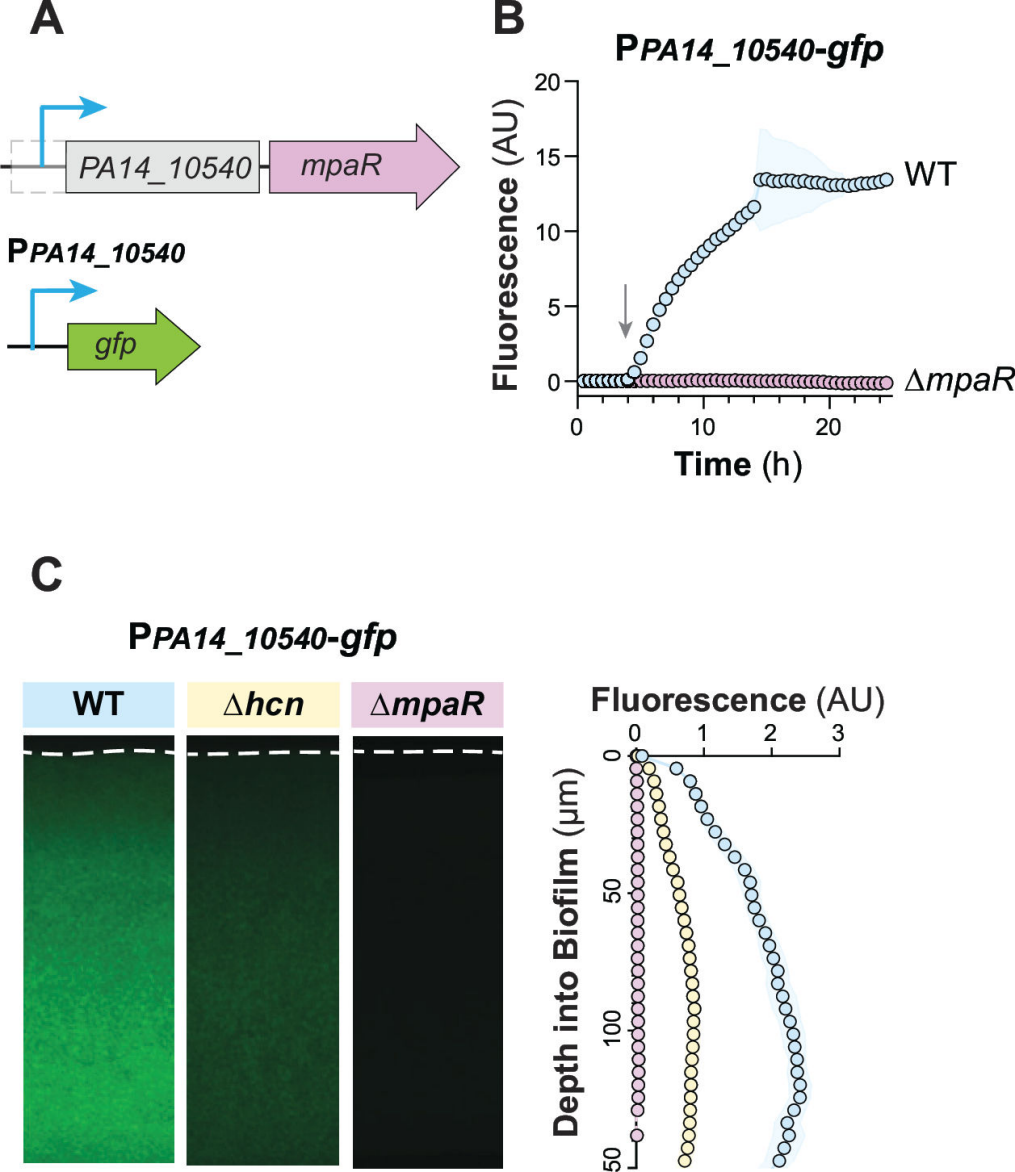
Cyanide-dependent and cyanide-independent expression of *mpaR* is driven by the promoter upstream of *PA14_10540*. (**A**) Schematics of the chromosomal region containing *PA14_10540-mpaR* and putative transcription start site (additional detail provided in the legend of Fig. 1A) and of the reporter construct. (**B**) Fluorescence of the indicated strains grown in liquid culture. Data represent average of three biological replicates, and shading indicates standard deviation. Arrow indicates the onset of stationary phase. (**C**) Representative thin-section images of the indicated strains, each containing the P*PA14_10540-gfp* reporter. Biofilms were grown for 3 days before harvesting and preparation of thin sections. For quantification, shown on the right, experiments were performed with biological triplicates, and shading indicates standard deviation.

Prior transcriptomic analysis carried out by Wurtzel et al. reveals the TSS for *PA14_10540* and indicates that *PA14_10540* and *mpaR* form a transcriptional unit (Fig. 6A) (23). The region upstream of the *PA14_10540* TSS contains a palindromic ATGNCAT motif (Fig. 4C), which would suggest control by MpaR (Fig. 4B) [we note that this experimentally determined TSS is located within the predicted ORF, whose start site may therefore be misannotated in The Pseudomonas Genome Database and BioCyc (43, 44)]. To test the requirement of MpaR for *PA14_10540* expression, we made a reporter construct (P*PA14_10540-gfp*) containing sequence spanning from 396 bp upstream to 30 bp downstream of the experimentally identified transcription start site (PA14 genomic locus: 908280) (23) (Fig. 6A). When grown as liquid cultures or biofilms, the P*PA14_10540* reporter strain showed high expression that was eliminated by *mpaR* deletion (Fig. 6B and C). Examining the effect of the ∆*hcn* mutation on these reporters, we found that the P*PA14_10540* reporter produced an intermediate level of cyanide-independent fluorescence (Fig. 6B and C). These results indicate that the *PA14_10540-mpaR* operon is autoregulated by MpaR and that it is expressed at low levels in the absence of cyanide and at high levels during cyanogenesis.

### Point-mutant phenotypes suggest that MpaR is activated by binding of pyridoxal phosphate or a related cofactor

**Fig 7:**
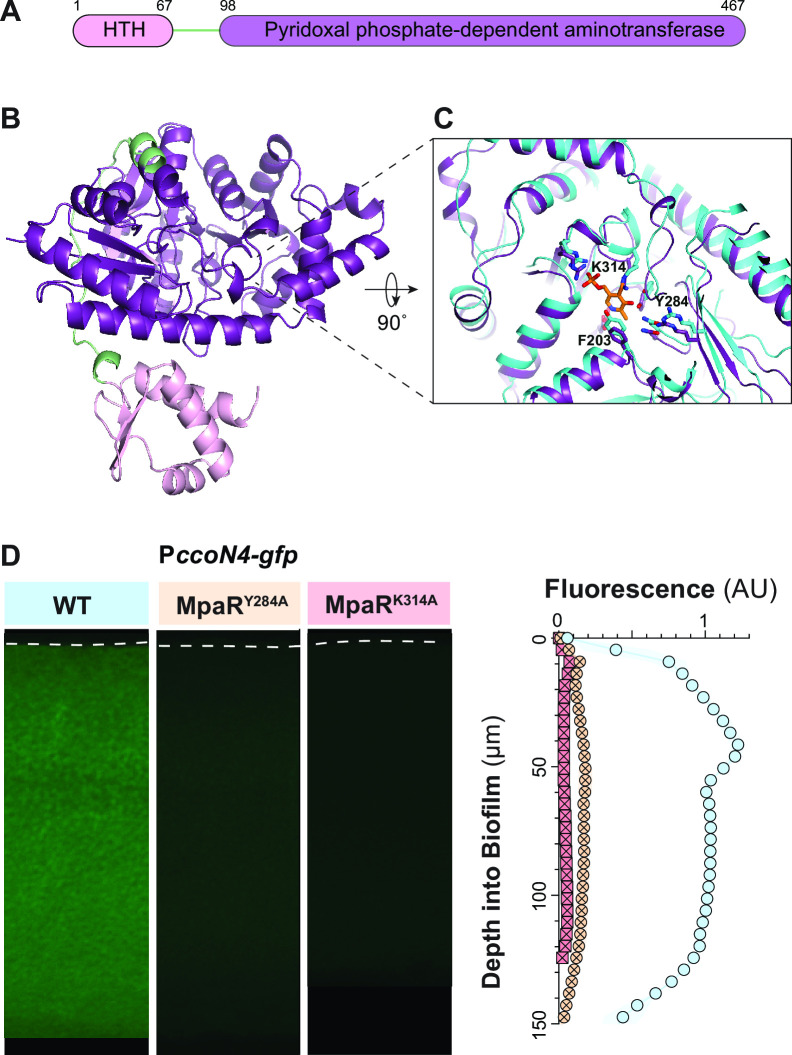
Residues predicted to interact with a pyridoxal phosphate cofactor are required for MpaR-driven expression of *ccoN4*. (**A**) Schematic of the MpaR protein showing putative DNA-binding (HTH) and cofactor (pyridoxal phosphate)-binding domains. Numbers correspond to the amino acid sequence of *P. aeruginosa* PA14 MpaR (45). (**B**) Structure of MpaR generated by Alphafold with domain coloration as shown in panel **A**. (**C**) Reoriented view of MpaR’s (purple) putative cofactor-binding pocket, overlaid on the structure for *Arabidopsis thaliana* LL-Diaminopimelate aminotransferase (PDB: 2Z1Z) (cyan). Tyrosine and lysine residues that extend into the pocket are annotated. (**D**) Representative thin-section images of the indicated strains, each containing the P*ccoN4-gfp* reporter construct. Biofilms were grown for 3 days before harvesting and preparation of thin sections. For quantification, shown on the right, experiments were performed with biological triplicates, and shading indicates standard deviation. Mass spectrometry quantification of protein abundance is shown in Fig. S7.

MpaR is a GntR family regulator with an N-terminal, DNA-binding helix-turn-helix (HTH) domain and a C-terminal domain that bears homology to pyridoxal phosphate (PLP)-dependent aminotransferases (Fig. 7A) (45). We hypothesized that binding of PLP or a similar cofactor could affect MpaR function and examined the structures of PLP-binding proteins that had been co-crystallized with PLP to identify conserved features. PLP-binding pockets contain a conserved lysine residue that covalently binds PLP as a Schiff base and a semi-conserved tyrosine whose ring coordinates the ring of PLP (46, 47). Using an MpaR structure predicted by Alphafold (Fig. 7B) (48, 49), we identified the corresponding residues in the putative PLP binding site: K314 and Y284 (Fig. 7C, shown overlaid with solved PLP-bound structure PDB: 2Z1Z). We then created strains in which each of these two residues were changed to alanines, in the P*ccoN4-gfp* reporter background. We found that the K314A mutation rendered P*ccoN4-gfp* activity undetectable, while a low level of P*ccoN4-gfp* activity remained in the Y284A mutant background (Fig. 7D). We also performed mass spectrometry analysis on extracts prepared from biofilms grown under the same conditions and found that these showed decreased abundances of MpaR mutant proteins (Fig. S7), consistent with the role of MpaR in regulating *PA14_10540-mpaR* (Fig. 6). MpaR’s PLP-dependent transferase domain is fold type I, and these domains generally bind PLP as homodimers with the active sites brought together (45, 50). Binding of PLP or a related cofactor may cause MpaR to dimerize in a way that promotes its activity as a transcription factor.

### MpaR and CcoN4 mitigate the effects of endogenous cyanide on biofilm development

**Fig 8:**
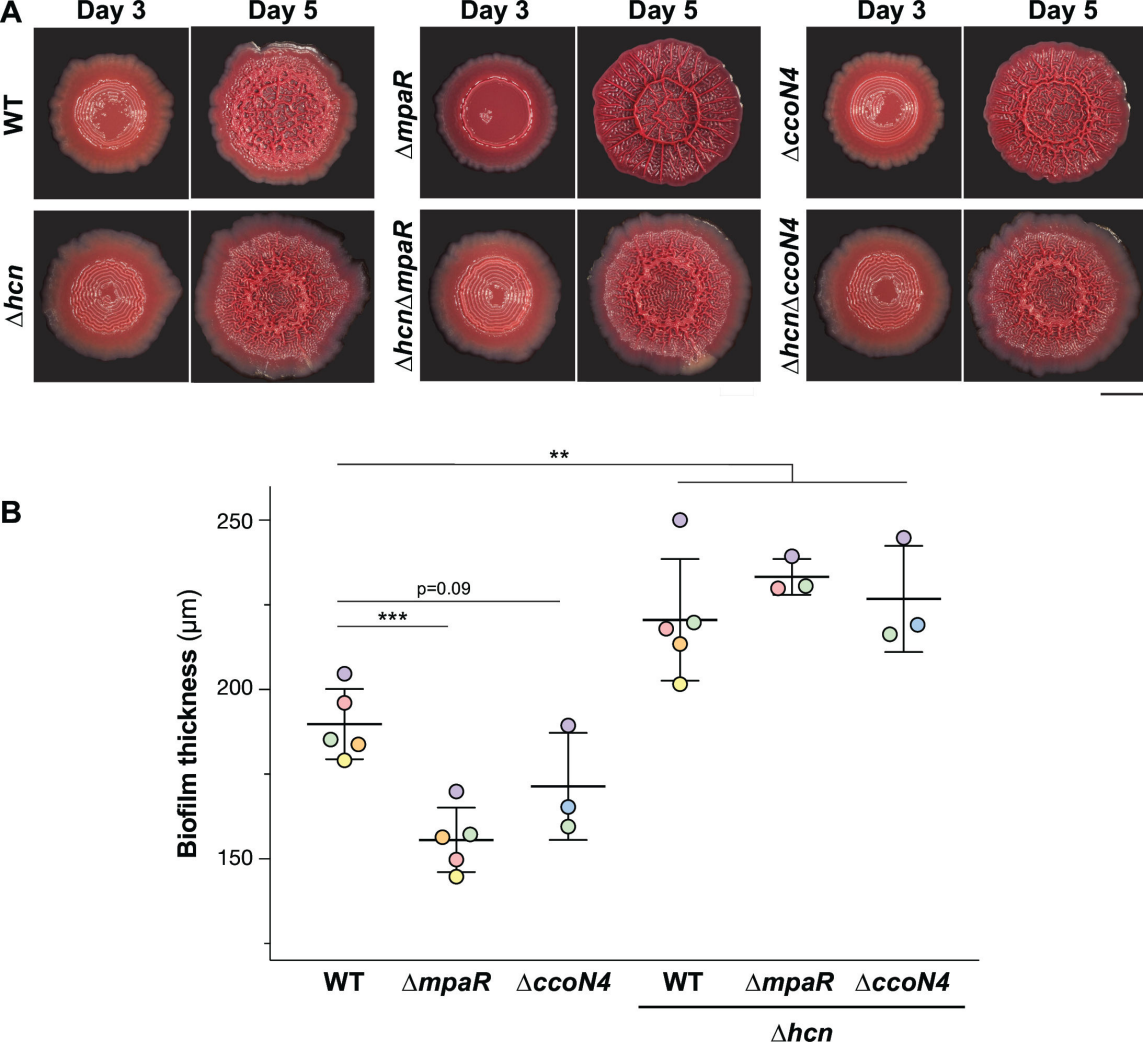
Biofilm development and thickness phenotypes indicate roles for MpaR and CcoN4 in cyanide self-resistance. (**A**) Representative images of biofilms grown in the colony biofilm morphology assay, shown after 3 and 5 days of growth. Scale bar is 5 mm. (**B**) Thickness of biofilms formed by the indicated strains. Biofilms were grown for 3 days before harvesting and preparation of thin sections. Each data point represents the average of biological replicates of the same strain that were grown on the same day. Data points are color coded to indicate values that were obtained on the same day. Each horizontal line represents the average of all values plotted for the indicated strain, and error bars represent standard deviation. *P*-values were calculated using unpaired, two-tailed *t* tests (**P < 0.01, ***P < 0.001). Complementation results for ∆*mpaR* and ∆*hcnB* strains are shown in Fig. S8.

The responses of *mpaR* and *ccoN4* to endogenous cyanide production in biofilms raised the question of whether these genes play roles in biofilm development. We first tested the *mpaR* deletion for effects on biofilm development using a colony morphology assay (51). We observed that ∆*mpaR* biofilms exhibit a hyperwrinkled phenotype with a distinctive inner ring (Fig. 8A). In addition, we used thin-section images of biofilms grown on dye-free media to measure biofilm thicknesses and found that ∆*mpaR* biofilms consistently measured approximately ~35 µm thinner than their WT counterparts; ∆*ccoN4* biofilms also showed a thinner phenotype, measuring ~25 µm thinner than those formed by the WT (Fig. 8B). Although alterations in phenazine production can affect colony biofilm morphogenesis, high-performance liquid chromatography (HPLC) analysis of phenazines produced by ∆*mpaR* biofilms showed only subtle changes relative to WT biofilms (Fig. S6), suggesting that ∆*mpaR* biofilm development phenotypes arise from other effects. Indeed, deletion of the *hcnABC* operon in the ∆*mpaR* and ∆*ccoN4* backgrounds yielded colony biofilms that more closely resembled WT and increased the thicknesses of biofilms formed by ∆*mpaR*, ∆*ccoN4*, and even the WT (Fig. 8B; Fig. S8). These results indicate that stress arising from the loss of MpaR and CcoN4 can be directly mitigated by removing stress caused by cyanide.

## DISCUSSION

*P. aeruginosa* releases chemically diverse small molecules that have physiological effects not only on other organisms, including hosts, but also on their producer (52). Among these, cyanide is noteworthy because it is volatile and a potent inhibitor of heme-copper oxidases, which are common components of mitochondrial, bacterial, and archaeal respiratory chains (21, 53, 54). *P. aeruginosa* cyanide production is particularly enigmatic because this bacterium is known for its proficient use of O_2_ as a terminal electron acceptor and because it contains six distinct operons encoding subunits of heme-copper oxidases (26, 55). Although *P. aeruginosa* can also produce a cyanide-insensitive (copper free) oxidase, growth and survival experiments indicate that this oxidase is not necessary for protection against endogenous cyanide under physiological conditions (22, 25). Counterintuitively, *P. aeruginosa* shows strong induction of *ccoN4*, encoding a heme-copper oxidase subunit, when treated with cyanide (20). Experiments with exogenously added cyanide suggest that, even though CcoN4-containing oxidases are predicted to be cyanide sensitive (19), this subunit acts synergistically with the cyanide-insensitive oxidase to confer cyanide resistance (20).

We set out to investigate the regulation of *ccoN4* expression in the context of *P. aeruginosa* PA14 toxin production, respiration, and biofilm physiology. *ccoN4* lies in a previously identified cluster of six genes that are induced by cyanide (20) and is immediately adjacent to the regulatory gene *mpaR* (39). Although we did not take an *in vitro* approach to studying MpaR’s interactions with its targets, the results of our genetic analyses allow us to make inferences about MpaR’s control of *ccoN4* and *PA14_10540-mpaR* expression. We confirmed that MpaR is required for *ccoN4* expression and that full induction of *ccoN4* requires endogenous cyanide production. Furthermore, we identified a short palindromic sequence (ATCTGAT) in the *ccoN4* promoter region that is necessary for this induction. An identical motif appears in the promoter regions of each of the three predicted operons within the so-called “cyanide-inducible gene cluster” (*ccoN4-ccoQ4*, *PA14_10540-mpaR*, and *nirA-PA14_10560*) at almost the exact same relative location (61, 62, and 62 bp upstream of their respective transcription start sites) (23) (Fig. 4C). Experiments with an engineered strain that constitutively produces MpaR revealed that cyanide production is required for full MpaR-dependent induction of *ccoN4* and not solely for induction of *mpaR*. The fact that the *PA14_10540* promoter contains the putative MpaR-binding motif suggests the existence of a positive feedback loop that amplifies the response to cyanide.

Prior transcriptomic studies, comparing WT *P. aeruginosa* to both a cyanide-null mutant (∆*hcnB*) and a ∆*mpaR* mutant, also allow us to make inferences regarding the regulatory effects of cyanide and MpaR. The genes in the “cyanide-inducible gene cluster” stood out among those in the cyanide stimulon due to their high level of induction (20) and also showed high changes in expression in the study comparing the WT and ∆*mpaR* transcriptomes (39) (Fig. S2). These findings suggest that cyanide affects transcription mainly through MpaR. Consistent with our observation that *ccoN4* expression occurs at low levels in the ∆*hcn* background but is abolished in the ∆*mpaR* background, the MpaR regulon contains genes that are not in the cyanide stimulon, and some of these show strong dependence on MpaR (20, 39), underscoring the possibility that MpaR exhibits both cyanide-independent and cyanide-dependent activity.

MpaR is predicted to contain an N-terminal helix-turn-helix DNA-binding domain and a C-terminal domain with homology to PLP-dependent aminotransferases, connected by a flexible 31-residue linker (44, 45). These characteristics suggest that MpaR is a member of the MocR subfamily within the GntR family of bacterial transcription factors (56). Although members of the MocR subfamily contain a domain with homology to PLP-dependent aminotransferases, studies have indicated that this domain is not enzymatically active and that in these proteins, binding of PLP and/or related compounds instead serves as a modulator of transcription factor function (57–60). In *Bacillus clausii* PdxR, for example, it has been shown that the binding of PLP to the C-terminal domain can affect the conformation of the N-terminal domain, thereby changing the protein’s affinity for distinct DNA-binding motifs (61). It should be noted that PLP, a critical cofactor in diverse metabolic pathways, reacts readily with cyanide to generate cyanohydrins (62, 63).

Given that we observe MpaR-dependent gene induction in response to cyanide, we hypothesize that MpaR is activated by binding of cyanide-modified PLP (i.e., the cyanohydrin form or PLP-CN), which triggers a conformational change in the N-terminal domain that promotes DNA binding. This mechanism of regulation would require that PLP and PLP-CN differentially affect MpaR’s conformation. To examine this possibility, we used Alphafold (48, 49) and PHYRE2 (64) to generate models of MpaR and Maestro (Schrödinger Suite) to perform Induced-Fit docking of PLP and PLP-CN in the MpaR binding site. (The results of this analysis are described in detail in File S1) Looking first at the full structure of MpaR, we overlaid a model of *apo* MpaR (generated with Alphafold) with a model of the MpaR holoenzyme (generated with PHYRE2 using PLP-bound PdxR as a template). This overlay indicates that binding of PLP and/or PLP-CN converts an alpha helix present in the *apo* form into a loop in the holo form. This helix/loop is preceded by a proline-rich stretch that is clustered near F114, the side chain of which faces the PLP/PLP-CN binding pocket. Related to this, our Induced-Fit docking analysis indicates a stronger binding of MpaR to PLP-CN than to PLP and that several shifts occur in the predicted interactions of side chain residues that face the binding site when PLP docking is compared to that of PLP-CN, including in those predicted for K314 (Fig. 7C and D) and F203. In particular, F203 forms a stronger interaction with the cyanohydrin form, which could cause a conformational change in F114 that is transduced to the proline-rich region and ultimately the DNA-binding domain.

Although organisms that produce cyanide, which include plants, fungi, cyanobacteria, and soil and plant-associated bacteria, risk self-poisoning, they can also benefit from its toxicity to predatory, pathogenic, or competing organisms (53, 65–68). *P. aeruginosa* cyanide production, which can yield concentrations of more than 100 µM [substantially higher than the level needed to kill eukaryotic cells (17, 69)], contributes to its interspecies competitive fitness and its pathogenicity and may also contribute to intraspecies, population-level fitness (70–74). Studies in plants and animals have also highlighted other physiological effects of cyanide production (75), including roles in regulation. Cyanide sensing in plant proteins may occur via chemical modification of cysteine (76), and although other bacterial cyanide-sensing proteins have yet to be identified, a few structures showing the effect of cyanide on ligand binding have been solved (77–79). Our findings suggest a new mechanism of cyanide-dependent control of gene expression in a cyanide-producing, pathogenic bacterium that could be targeted in therapeutic approaches that weaken *P. aeruginosa’s* self-resistance and toxicity. Moreover, our results point to a possible strategy for the quantification of cyanide that relies on PLP-binding transcription factors, which could be exploited in diverse industrial settings (80–82).

## MATERIALS AND METHODS

### Strains and growth conditions

All bacterial strains used in this study are listed in Table S1. For liquid culture growth, single colonies of *Pseudomonas aeruginosa* UCBPP-PA14 (83) were used to inoculate lysogeny broth (LB; 1% tryptone, 1% NaCl, 0.5% yeast extract) (84) or 1% tryptone, as described, and shaken at 37°C, 200 rpm. Generally, liquid precultures were used as inocula for experiments. Biological replicates came from separate clonal source colonies on a streaked agar plate.

### TTC reduction assay

Precultures were grown for 14–16 hours, subcultured 1:100, and grown to an OD 500 nm of ~0.5. Five microliters were spotted onto 60 mL of 1% tryptone, 1% agar (Teknova, A7777) containing 0.004% (wt/vol) TTC [2,3,5-triphenyl-tetrazolium chloride (Sigma-Aldrich, T8877)] in 10 cm × 10 cm × 1.5 cm plates. Plates were incubated for 1 day in the dark at 25°C with >90% humidity and imaged using a flatbed scanner (Epson, E11000XL-GA). TTC reduction was quantified by measuring the average saturation in the red hue for all pixels in the colony biofilm area.

### Construction of fluorescent reporter strains

All plasmids used in this study are listed in Table S2. To construct reporter strains, promoter regions of varying length or with nucleotide changes (as specified in the strain name and in the text) were amplified from the PA14 genome using primers listed in Table S3 and inserted upstream of the coding sequence of *gfp* using SpeI and XhoI sites in the MCS of the pLD2722 vector. Plasmids were transformed into *E. coli* UQ950 cells and verified by sequencing. Verified plasmids were introduced into PA14 using biparental conjugation with *E. coli* S17-1. Single recombinants were selected on agar plates with M9 minimal medium (47.8 mM Na_2_HPO_4_7H_2_O, 2 mM KH_2_PO_4_, 8.6 mM NaCl, 18.6 mM NH_4_Cl, 1 mM MgSO_4_, 0.1 mM CaCl_2_, 20 mM sodium citrate dihydrate, 1.5% agar) containing 70 µg/mL gentamicin. The plasmid backbone was resolved out of PA14 using Flp-FRT recombination using the pFLP2 plasmid (85) and selection on M9 minimal medium agar plates containing 300 µg/mL carbenicillin. Strains were cured of the pFLP2 plasmid by streaking on LB agar plates without NaCl with 10% (wt/vol) sucrose. The presence of *gfp* in final clones was confirmed by PCR.

Shuffled reporters were constructed using an overlap PCR in which two fragments were generated flanking the region to be shuffled with the shuffled sequence introduced with the primers. Then, the overlap PCR was performed such that one single fragment was generated from the two fragments. Fragment was inserted into pLD2722 using SpeI and XhoI digest sites and introduced into PA14 as above.

### Construction of deletion mutant strains

All plasmids used in this study are listed in Table S2. Markerless deletion mutants in *Pseudomonas aeruginosa* UCBPP-PA14 were made by amplifying ~1 kb of flanking sequence on each side of the deletion region using the primers listed in Table S3 and inserting flanks into pMQ30 plasmid through gap repair cloning in *S. cerevisiae* InvSc1 (86). Plasmids were transformed into *E. coli* UQ950, verified by sequencing, and introduced into PA14 using biparental conjugation with *E. coli* WM3064. Single recombinants were selected on LB agar plates containing 100 µg/mL gentamicin. Double recombinants were selected on LB without NaCl with 10% (wt/vol) sucrose. Deletions were verified by PCR. Combinatorial mutants were made using a single deletion as PA14 parental background. Complements were made as described above by amplifying the flanking regions and the coding sequence together and introduced into the deletion background. Point mutants were created as described for deletion mutants with the exception that the initial primers introduced the specified codon change and included the entire coding region and ~1 kb of flanking sequence. Constructs were then introduced into the deletion strain and checked for “complementation.”

### Construction of MpaR overexpression strains

The coding region of MpaR was amplified using primers LD4387 and LD4388, and the *rrnB* terminator was amplified from pAKN69 using primers LD4389 and LD4077 such that the two fragments had ~20-bp overlapping sequence similarity. An overlap PCR was performed using LD4387 and LD4077 and the two fragments (~75 ng each) as template. The resulting fragment (MpaR coding region with a terminator) was restriction digest cloned into pAKN69 using SphI and NheI restriction enzymes. The resulting plasmid was transformed into *E. coli* WM3064, verified by sequencing, and introduced into PA14 using biparental conjugation with *E. coli* WM3064. Single recombinants were selected on LB agar plates containing 100 µg/mL gentamicin. Plasmid backbones were not resolved out.

### Colony biofilm morphology assay

Precultures grown for 14–16 hours were subcultured 1:100 and grown until OD 500 nm of ~0.5. 10 µL was spotted onto 60 mL of colony morphology medium [1% tryptone, 1% agar (Teknova, A7777)] containing 40 µg/mL Congo red dye (VWR, AAAB24310-14) and 20 µg/mL Coomassie blue dye (VWR, EM-3300)] in a 10 cm × 10 cm × 1.5 cm plates. Plates were incubated for up to 5 days at 25°C with >90% humidity and imaged using a flatbed scanner (Epson, E11000XL-GA).

### Biofilm thin sectioning

Sectioning was performed as previously described (87). Briefly, precultures grown for 14–16 hours were subcultured 1:100 and grown until OD 500 nm of ~0.5. Five microliters were spotted onto 45 mL/15 mL bilayer 1% tryptone 1% agar (Teknova, A7777) square 10 cm × 10 cm × 1.5 cm plates and incubated for 72 hours at 25°C. Biofilms were overlaid with 15 mL of 1% molten agar, allowed to solidify for 5–10 minutes, and then excised by lifting from the bottom layer into histocassettes where they were fixed in 4% paraformaldehyde in phosphate-buffered saline (PBS) (pH 7.2) for 24 hours. Samples were then washed in a series of increasing ethanol concentrations (25%, 50%, 70%, 95% ethanol in PBS, three times in 100% ethanol) followed by clearing with three washes in Histo-Clear II (National Diagnostics; Fisher Scientific HS-202) and then infiltrated with molten paraffin wax (Leica Biosystems, 39601006) . Samples were then embedded in paraffin wax in rectangular wax molds and allowed to solidify for 2–3 hours. Excess wax was removed from the sample blocks, and the center of the biofilm was sectioned in 10-µm steps using an automatic microtome (Thermo Fisher Scientific, 905200ER). Sections were floated in water and mounted on a pre-charged glass slide. Slides air dried overnight then heat fixed at 45°C for 30 minutes. Once cooled, the slides were rehydrated in PBS by reversing the dehydration steps listed above. Sections were mounted with Prolong Diamond Antifade Mountant (Invitrogen; Thermo Fisher Scientific, P36965) and covered with coverslips. Coverslips were sealed with quick-dry nail polish and imaged on a ZEISS Axio Zoom.V16. Non-fluorescent parental strains were used as a control for colony auto-fluorescence. In the graphs shown, fluorescent signal has been corrected for the respective parental background fluorescence.

### Liquid culture growth and reporter-strain fluorescence assays

Overnight precultures in biological triplicate were diluted 1:100 into 1% tryptone in 96-well, black-sided, clear-bottomed plate (Greiner Bio-One; Millipore Sigma, M5811) and incubated at 37°C with continuous shaking on the medium setting in a Biotek Synergy H1 plate reader. Growth and promoter activity were assessed by taking OD readings at 500 nm and fluorescence readings at excitation and emission wavelengths of 480 nm and 510 nm, respectively, every 30 minutes for 24 hours.

### Cyanide-dependent induction assay

Precultures were grown for 14–16 hours, then subcultured 1:100, and grown to an OD 500 nm of ~0.5. Five microliters of the cultures were spotted ~1 cm apart onto 60 mL of 1% tryptone, 1% agar (Teknova, A7777) in a 10 cm × 10 cm × 1.5 cm plates. Plates were incubated for up to 3 days at 25°C with >90% humidity and imaged using a ZEISS Axio Zoom.V16. No additional cyanide was added to the medium.

### High-performance liquid chromatography

Precultures grown for 14–16 hours were subcultured 1:100 and grown until OD 500 nm of ~0.5. Ten microliters were spotted onto 4 mL of 1% tryptone, 1% agar (Teknova, A7777) in 30-mm circular petri plates. Plates were incubated for 3 days in the dark at 25°C with >90% humidity. The entire biofilm and agar were harvested by submerging in 5 mL of 100% methanol and rocking overnight at room temperature. The following day, the methanol extracts were filtered through Costar 0.22-µm cellulose acetate Spin-X centrifuge tube filters (Fisher Scientific, 07200386); then, 200 µL was loaded into HPLC vials for analysis using high-performance liquid chromatography (Agilent 1100 HPLC system) as previously described (88, 89) and by comparing sample peaks to peaks of pure phenazine standards run as controls. The area under each peak was integrated and used to determine the concentration of each phenazine.

### Sample preparation for Liquid Chromatography with Tandem Mass Spectrometry (LC-MS/MS) measurements

Precultures of *Pseudomonas aeruginosa* grown for 14–16 hours were subcultured 1:100 and grown until OD 500 nm of ~0.5. Five microliters were spotted onto 60-mL 1% tryptone 1% agar (Teknova, A7777) square 10 cm × 10 cm × 1.5 cm plates and incubated for 72 hours at 25°C. Biofilms were collected into 1 mL of PBS and were physically disrupted by pipetting. Pellets were collected and washed twice more with PBS before resuspending in 500-µL Mass Spec Lysis Buffer (8 M urea, 75 mM NaCl, 50 mM Tris pH 8.0, 1 mM EDTA). Samples were sonicated on ice using the microtip of a Branson Sonifier 250 (50% duty cycle 50% output) for 90 seconds (30-second sonication, 1-minute rest three times). Samples were clarified by centrifugation for 20,000 × *g* for 5 minutes, and the protein content of the supernatant was measured by Qubit. Forty micrograms of protein for each sample were reduced with a final concentration of 5 mM dithriothreitol (DTT) for 45 minutes at room temperature and then alkylated with a final concentration of 10 mM iodoacetamide (IAA) for 45 minutes in the dark at room temperature. Samples were diluted with 50 mM Tris (pH 8.0) such that the final concentration of urea was less than 2 M. Samples were digested overnight with 0.4 µg trypsin (Promega, V5113) for a 1:100 enzyme to protein ratio. After 16 hours of digestion, samples were acidified with 1% formic acid (final concentration). Tryptic peptides were desalted on C18 StageTips according to reference (90) and evaporated to dryness in a vacuum concentrator. Dried peptides were then reconstituted in 3% acetonitrile/0.2% formic acid to a final concentration of 0.5 µg/µL.

### LC-MS/MS analysis on a Q-Exactive HF

About 1 µg of total peptides was analyzed on a Waters M-Class UPLC using a 15-cm Ionopticks Aurora Elite SX nanoflow UHPLC column (C18 1.7-µm stationary phase) Thermo Fisher Scientific Orbitrap Q Exactive HF mass spectrometer. Peptides were separated at a flow rate of 400 nL/min with a 90-minute gradient, including sample loading and column equilibration times. Data were acquired in data independent mode using Xcalibur software. MS1 spectra were measured with a resolution of 120,000, and a mass range from 350 to 1650 m/z. MS2 spectra were measured in 23 segment windows per MS1, each with an isolation window width of 56 m/z (0.5 m/z overlap with the neighboring window), a resolution of 30,000, an AGC target of 3e6, and a stepped collision energy of 22.5, 25, 27.5.

All raw data were analyzed with SpectroNaut software version 18.0 (Biognosys) using a directDIA method based on a UniProt *Pseudomonas aeruginosa* database (UP000000653), performed with the “BGS factory settings” including the following parameters: oxidation of methionine and protein N-terminal acetylation as variable modifications; carbamidomethylation as fixed modification; trypsin/P as the digestion enzyme. For identification, we applied a maximum FDR of 1% separately on protein and peptide level. “Cross run normalization” was activated. This gave intensity values for a total of 3,024 protein groups across all samples and replicates. “PG.Quantity” (normalized across samples) values were used for all subsequent analyses.

## SUPPLEMENTAL MATERIAL

File S1 mbio.02926-23-s0001.pdf Accompaniment to Figure 7.

Supplemental Figures and Tables mbio.02926-23-s0002.pdf Figures 1-8 and Tables 1-3.

ASM does not own the copyrights to Supplemental Material that may be linked to, or accessed through, an article. The authors have granted ASM a non-exclusive, world-wide license to publish the Supplemental Material files. Please contact the corresponding author directly for reuse.

## References

[1] Evans CR, Kempes CP, Price-Whelan A, Dietrich LEP. 2020. Metabolic heterogeneity and cross-feeding in bacterial multicellular systems. Trends Microbiol 28:732–743. doi:10.1016/j.tim.2020.03.008 32781027 PMC7419710

[2] Jo J, Price-Whelan A, Dietrich LEP. 2022. Gradients and consequences of heterogeneity in biofilms. Nat Rev Microbiol 20:593–607. doi:10.1038/s41579-022-00692-2 35149841 PMC9590228

[3] van Gestel J, Vlamakis H, Kolter R, Ghannoum M, Parsek M, Whiteley M, Mukherjee P. 2015. Division of labor in biofilms: the ecology of cell differentiation. Microbiol Spectr 3. doi:10.1128/microbiolspec.MB-0002-2014 26104716

[4] Williamson KS, Richards LA, Perez-Osorio AC, Pitts B, McInnerney K, Stewart PS, Franklin MJ. 2012. Heterogeneity in Pseudomonas aeruginosa biofilms includes expression of ribosome hibernation factors in the antibiotic-tolerant subpopulation and hypoxia-induced stress response in the metabolically active population. J Bacteriol 194:2062–2073. doi:10.1128/JB.00022-12 22343293 PMC3318454

[5] Stewart PS, Franklin MJ. 2008. Physiological heterogeneity in biofilms. Nat Rev Microbiol 6:199–210. doi:10.1038/nrmicro1838 18264116

[6] Armstrong AV, Stewart-Tull DE. 1971. The site of the activity of extracellular products of Pseudomonas aeruginosa in the electron-transport chain in mammalian cell respiration. J Med Microbiol 4:263–270. doi:10.1099/00222615-4-2-263 4328182

[7] Hazan R, Que YA, Maura D, Strobel B, Majcherczyk PA, Hopper LR, Wilbur DJ, Hreha TN, Barquera B, Rahme LG. 2016. Auto poisoning of the respiratory chain by a quorum-sensing-regulated molecule favors biofilm formation and antibiotic tolerance. Curr Biol 26:195–206. doi:10.1016/j.cub.2015.11.056 26776731 PMC4729643

[8] Voggu L, Schlag S, Biswas R, Rosenstein R, Rausch C, Götz F. 2006. Microevolution of cytochrome bd oxidase in staphylococci and its implication in resistance to respiratory toxins released by Pseudomonas. J Bacteriol 188:8079–8086. doi:10.1128/JB.00858-06 17108291 PMC1698191

[9] Ciemniecki JA, Newman DK. 2023. NADH dehydrogenases are the predominant phenazine reductases in the electron transport chain of Pseudomonas aeruginosa. Mol Microbiol 119:560–573. doi:10.1111/mmi.15049 36840394 PMC11129870

[10] Pessi G, Haas D. 2000. Transcriptional control of the hydrogen cyanide biosynthetic genes hcnABC by the anaerobic regulator ANR and the quorum-sensing regulators LasR and RhlR in Pseudomonas aeruginosa. J Bacteriol 182:6940–6949. doi:10.1128/JB.182.24.6940-6949.2000 11092854 PMC94819

[11] Sakhtah H, Price-Whelan A, Dietrich LEP. 2013. Regulation of phenazine biosynthesis, p 19–42. In Chincholkar S, Thomashow L (ed), Microbial phenazines: biosynthesis, agriculture and health. Springer, Berlin Heidelberg, Berlin, Heidelberg.

[12] Wang Y, Kern SE, Newman DK. 2010. Endogenous phenazine antibiotics promote anaerobic survival of Pseudomonas aeruginosa via extracellular electron transfer. J Bacteriol 192:365–369. doi:10.1128/JB.01188-09 19880596 PMC2798253

[13] Glasser NR, Kern SE, Newman DK. 2014. Phenazine redox cycling enhances anaerobic survival in Pseudomonas aeruginosa by facilitating generation of ATP and a proton-motive force. Mol Microbiol 92:399–412. doi:10.1111/mmi.12566 24612454 PMC4046897

[14] Jo J, Cortez KL, Cornell WC, Price-Whelan A, Dietrich LE. 2017. An orphan cbb_3_-type cytochrome oxidase subunit supports Pseudomonas aeruginosa biofilm growth and virulence. Elife 6. doi:10.7554/eLife.30205 PMC5697931 29160206

[15] Blumer C, Haas D. 2000. Mechanism, regulation, and ecological role of bacterial cyanide biosynthesis. Arch Microbiol 173:170–177. doi:10.1007/s002039900127 10763748

[16] Sanderson K, Wescombe L, Kirov SM, Champion A, Reid DW. 2008. Bacterial cyanogenesis occurs in the cystic fibrosis lung. Eur Respir J 32:329–333. doi:10.1183/09031936.00152407 18480103

[17] Ryall B, Davies JC, Wilson R, Shoemark A, Williams HD. 2008. Pseudomonas aeruginosa, cyanide accumulation and lung function in CF and non-CF bronchiectasis patients. Eur Respir J 32:740–747. doi:10.1183/09031936.00159607 18480102

[18] Mitchell R, Brown S, Mitchell P, Rich PR. 1992. Rates of cyanide binding to the catalytic intermediates of mammalian cytochrome c oxidase, and the effects of cytochrome c and poly(L-lysine). Biochim Biophys Acta 1100:40–48. doi:10.1016/0005-2728(92)90124-k 1314665

[19] Hirai T, Osamura T, Ishii M, Arai H. 2016. Expression of multiple cbb_3_ cytochrome c oxidase Isoforms by combinations of multiple Isosubunits in Pseudomonas aeruginosa. Proc Natl Acad Sci U S A 113:12815–12819. doi:10.1073/pnas.1613308113 27791152 PMC5111723

[20] Frangipani E, Pérez-Martínez I, Williams HD, Cherbuin G, Haas D. 2014. A novel cyanide-inducible gene cluster helps protect Pseudomonas aeruginosa from cyanide. Environ Microbiol Rep 6:28–34. doi:10.1111/1758-2229.12105 24596260

[21] Cooper CE, Brown GC. 2008. The inhibition of mitochondrial cytochrome oxidase by the gases carbon monoxide, nitric oxide, hydrogen cyanide and hydrogen sulfide: chemical mechanism and physiological significance. J Bioenerg Biomembr 40:533–539. doi:10.1007/s10863-008-9166-6 18839291

[22] Zlosnik JEA, Tavankar GR, Bundy JG, Mossialos D, O’Toole R, Williams HD. 2006. Investigation of the physiological relationship between the cyanide-insensitive oxidase and cyanide production in Pseudomonas aeruginosa. Microbiology 152:1407–1415. doi:10.1099/mic.0.28396-0 16622057

[23] Wurtzel O, Yoder-Himes DR, Han K, Dandekar AA, Edelheit S, Greenberg EP, Sorek R, Lory S, Deretic V. 2012. The single-nucleotide resolution transcriptome of Pseudomonas aeruginosa grown in body temperature. PLoS Pathog 8:e1002945. doi:10.1371/journal.ppat.1002945 23028334 PMC3460626

[24] Buschmann S, Warkentin E, Xie H, Langer JD, Ermler U, Michel H. 2010. The structure of cbb_3_ cytochrome oxidase provides insights into proton pumping. Science 329:327–330. doi:10.1126/science.1187303 20576851

[25] Arai H, Kawakami T, Osamura T, Hirai T, Sakai Y, Ishii M. 2014. Enzymatic characterization and in vivo function of five terminal oxidases in Pseudomonas aeruginosa. J Bacteriol 196:4206–4215. doi:10.1128/JB.02176-14 25182500 PMC4248849

[26] Kawakami T, Kuroki M, Ishii M, Igarashi Y, Arai H. 2010. Differential expression of multiple terminal oxidases for aerobic respiration in Pseudomonas aeruginosa. Environ Microbiol 12:1399–1412. doi:10.1111/j.1462-2920.2009.02109.x 19930444

[27] Alvarez-Ortega C, Harwood CS. 2007. Responses of Pseudomonas aeruginosa to low oxygen indicate that growth in the cystic fibrosis lung is by aerobic respiration. Mol Microbiol 65:153–165. doi:10.1111/j.1365-2958.2007.05772.x 17581126 PMC4157922

[28] Comolli JC, Donohue TJ. 2004. Differences in two Pseudomonas aeruginosa cbb_3_ cytochrome Oxidases. Mol Microbiol 51:1193–1203. doi:10.1046/j.1365-2958.2003.03904.x 14763990

[29] Ducluzeau A-L, Ouchane S, Nitschke W. 2008. The cbb_3_ oxidases are an ancient innovation of the domain bacteria. Mol Biol Evol 25:1158–1166. doi:10.1093/molbev/msn062 18353797

[30] de Gier JW, Schepper M, Reijnders WN, van Dyck SJ, Slotboom DJ, Warne A, Saraste M, Krab K, Finel M, Stouthamer AH, van Spanning RJ, van der Oost J. 1996. Structural and functional analysis of aa3-type and cbb_3_-type cytochrome C oxidases of paracoccus denitrificans reveals significant differences in proton-pump design. Mol Microbiol 20:1247–1260. doi:10.1111/j.1365-2958.1996.tb02644.x 8809776

[31] Zufferey R, Preisig O, Hennecke H, Thöny-Meyer L. 1996. Assembly and function of the cytochrome cbb_3_ oxidase subunits in bradyrhizobium japonicum. J Biol Chem 271:9114–9119. doi:10.1074/jbc.271.15.9114 8621562

[32] Rich PR, Mischis LA, Purton S, Wiskich JT. 2001. The sites of interaction of triphenyltetrazolium chloride with mitochondrial respiratory chains. FEMS Microbiol Lett 202:181–187. doi:10.1111/j.1574-6968.2001.tb10801.x 11520612

[33] Sana TG, Lomas R, Gimenez MR, Laubier A, Soscia C, Chauvet C, Conesa A, Voulhoux R, Ize B, Bleves S, DiRita VJ. 2019. Differential modulation of quorum sensing signaling through QslA in Pseudomonas aeruginosa strains PAO1 and PA14. J Bacteriol 201:e00362-19. doi:10.1128/JB.00362-19 31405911 PMC6779463

[34] Grace A, Sahu R, Owen DR, Dennis VA. 2022. Pseudomonas aeruginosa reference strains PAO1 and PA14: a genomic, phenotypic, and therapeutic review. Front Microbiol 13:1023523. doi:10.3389/fmicb.2022.1023523 36312971 PMC9607943

[35] Castric PA. 1975. Hydrogen cyanide, a secondary metabolite of Pseudomonas aeruginosa. Can J AMicrobiol 21:613–618. doi:10.1139/m75-088 164997

[36] Laville J, Blumer C, Von Schroetter C, Gaia V, Défago G, Keel C, Haas D. 1998. Characterization of the hcnABC gene cluster encoding hydrogen cyanide synthase and anaerobic regulation by ANR in the strictly aerobic biocontrol agent Pseudomonas fluorescens CHA0. J Bacteriol 180:3187–3196. doi:10.1128/JB.180.12.3187-3196.1998 9620970 PMC107821

[37] Fenn S, Dubern J-F, Cigana C, De Simone M, Lazenby J, Juhas M, Schwager S, Bianconi I, Döring G, Elmsley J, Eberl L, Williams P, Bragonzi A, Cámara M, Goldberg JB. 2021. NirA is an alternative nitrite reductase from Pseudomonas aeruginosa with potential as an antivirulence target. mBio 12. doi:10.1128/mBio.00207-21 PMC8092218 33879591

[38] Marckmann D, Trasnea P-I, Schimpf J, Winterstein C, Andrei A, Schmollinger S, Blaby-Haas CE, Friedrich T, Daldal F, Koch H-G. 2019. The cbb_3_-type cytochrome oxidase assembly factor CcoG is a widely distributed cupric reductase. Proc Natl Acad Sci U S A 116:21166–21175. doi:10.1073/pnas.1913803116 31570589 PMC6800367

[39] Wang T, Qi Y, Wang Z, Zhao J, Ji L, Li J, Cai Z, Yang L, Wu M, Liang H. 2020. Coordinated regulation of anthranilate metabolism and bacterial virulence by the GntR family regulator MpaR in Pseudomonas aeruginosa. Mol Microbiol 114:857–869. doi:10.1111/mmi.14584 32748556

[40] Trunk K, Benkert B, Quäck N, Münch R, Scheer M, Garbe J, Jänsch L, Trost M, Wehland J, Buer J, Jahn M, Schobert M, Jahn D. 2010. Anaerobic adaptation in Pseudomonas aeruginosa: definition of the Anr and Dnr regulons. Environ Microbiol 12:1719–1733. doi:10.1111/j.1462-2920.2010.02252.x 20553552

[41] Déziel E, Gopalan S, Tampakaki AP, Lépine F, Padfield KE, Saucier M, Xiao G, Rahme LG. 2005. The contribution of MvfR to Pseudomonas aeruginosa pathogenesis and quorum sensing circuitry regulation: multiple quorum sensing-regulated genes are modulated without affecting lasRI, rhlRI or the production of N-acyl-L-homoserine lactones. Mol Microbiol 55:998–1014. doi:10.1111/j.1365-2958.2004.04448.x 15686549

[42] Lanzer M, Bujard H. 1988. Promoters largely determine the efficiency of repressor action. Proc Natl Acad Sci U S A 85:8973–8977. doi:10.1073/pnas.85.23.8973 3057497 PMC282639

[43] Karp PD, Billington R, Caspi R, Fulcher CA, Latendresse M, Kothari A, Keseler IM, Krummenacker M, Midford PE, Ong Q, Ong WK, Paley SM, Subhraveti P. 2019. The BioCyc collection of microbial genomes and metabolic pathways. Brief Bioinform 20:1085–1093. doi:10.1093/bib/bbx085 29447345 PMC6781571

[44] Winsor GL, Griffiths EJ, Lo R, Dhillon BK, Shay JA, Brinkman FSL. 2016. Enhanced annotations and features for comparing thousands of Pseudomonas genomes in the Pseudomonas genome database. Nucleic Acids Res 44:D646–D653. doi:10.1093/nar/gkv1227 26578582 PMC4702867

[45] Paysan-Lafosse T, Blum M, Chuguransky S, Grego T, Pinto BL, Salazar GA, Bileschi ML, Bork P, Bridge A, Colwell L, et al.. 2023. Interpro in 2022. Nucleic Acids Res 51:D418–D427. doi:10.1093/nar/gkac993 36350672 PMC9825450

[46] Krupka HI, Huber R, Holt SC, Clausen T. 2000. Crystal structure of cystalysin from treponema denticola: a pyridoxal 5’-phosphate-dependent protein acting as a haemolytic enzyme. EMBO J 19:3168–3178. doi:10.1093/emboj/19.13.3168 10880431 PMC313955

[47] Tramonti A, Ghatge MS, Babor JT, Musayev FN, di Salvo ML, Barile A, Colotti G, Giorgi A, Paredes SD, Donkor AK, Al Mughram MH, de Crécy-Lagard V, Safo MK, Contestabile R. 2022. Characterization of the Escherichia coli pyridoxal 5’-phosphate homeostasis protein (YggS): role of lysine residues in PLP binding and protein stability. Protein Sci 31:e4471. doi:10.1002/pro.4471 36218140 PMC9601805

[48] Jumper J, Evans R, Pritzel A, Green T, Figurnov M, Ronneberger O, Tunyasuvunakool K, Bates R, Žídek A, Potapenko A, et al.. 2021. Highly accurate protein structure prediction with AlphaFold. Nature 596:583–589. doi:10.1038/s41586-021-03819-2 34265844 PMC8371605

[49] Varadi M, Anyango S, Deshpande M, Nair S, Natassia C, Yordanova G, Yuan D, Stroe O, Wood G, Laydon A, et al.. 2022. AlphaFold protein structure database: massively expanding the structural coverage of protein-sequence space with high-accuracy models. Nucleic Acids Res 50:D439–D444. doi:10.1093/nar/gkab1061 34791371 PMC8728224

[50] Kirsch JF, Eichele G, Ford GC, Vincent MG, Jansonius JN, Gehring H, Christen P. 1984. Mechanism of action of aspartate aminotransferase proposed on the basis of its spatial structure. J Mol Biol 174:497–525. doi:10.1016/0022-2836(84)90333-4 6143829

[51] Friedman L, Kolter R. 2004. Genes involved in matrix formation in Pseudomonas aeruginosa PA14 biofilms. Mol Microbiol 51:675–690. doi:10.1046/j.1365-2958.2003.03877.x 14731271

[52] Perry EK, Meirelles LA, Newman DK. 2022. From the soil to the clinic: the impact of microbial secondary metabolites on antibiotic tolerance and resistance. Nat Rev Microbiol 20:129–142. doi:10.1038/s41579-021-00620-w 34531577 PMC8857043

[53] Létoffé S, Wu Y, Darch SE, Beloin C, Whiteley M, Touqui L, Ghigo J-M, Kline KA. 2022. Pseudomonas aeruginosa production of hydrogen cyanide leads to airborne control of Staphylococcus aureus growth in biofilm and in vivo lung environments. mBio 13:e0215422. doi:10.1128/mbio.02154-22 36129311 PMC9600780

[54] Pei J, Li W, Kinch LN, Grishin NV. 2014. Conserved evolutionary units in the heme-copper oxidase superfamily revealed by novel homologous protein families. Protein Sci 23:1220–1234. doi:10.1002/pro.2503 24931479 PMC4243994

[55] Williams HD, Zlosnik JEA, Ryall B. 2007. Oxygen, cyanide and energy generation in the cystic fibrosis pathogen Pseudomonas aeruginosa. Adv Microb Physiol 52:1–71. doi:10.1016/S0065-2911(06)52001-6 17027370

[56] Rigali S, Derouaux A, Giannotta F, Dusart J. 2002. Subdivision of the helix-turn-helix GntR family of bacterial regulators in the FadR, HutC, MocR, and YtrA subfamilies. J Biol Chem 277:12507–12515. doi:10.1074/jbc.M110968200 11756427

[57] Tramonti A, Nardella C, di Salvo ML, Pascarella S, Contestabile R. 2018. The MocR-like transcription factors: pyridoxal 5’-phosphate-dependent regulators of bacterial metabolism. FEBS J 285:3925–3944. doi:10.1111/febs.14599 29974999

[58] Suvorova IA, Rodionov DA. 2016. Comparative genomics of pyridoxal 5’-phosphate-dependent transcription factor regulons in bacteria. Microb Genom 2:e000047. doi:10.1099/mgen.0.000047 28348826 PMC5320631

[59] Edayathumangalam R, Wu R, Garcia R, Wang Y, Wang W, Kreinbring CA, Bach A, Liao J, Stone TA, Terwilliger TC, Hoang QQ, Belitsky BR, Petsko GA, Ringe D, Liu D. 2013. Crystal structure of Bacillus subtilis GabR, an autorepressor and transcriptional activator of gabT. Proc Natl Acad Sci U S A 110:17820–17825. doi:10.1073/pnas.1315887110 24127574 PMC3816445

[60] Wu R, Sanishvili R, Belitsky BR, Juncosa JI, Le HV, Lehrer HJS, Farley M, Silverman RB, Petsko GA, Ringe D, Liu D. 2017. PLP and GABA trigger GabR-mediated transcription regulation in Bacillus subtilis via external aldimine formation. Proc Natl Acad Sci U S A 114:3891–3896. doi:10.1073/pnas.1703019114 28348215 PMC5393194

[61] Tramonti A, Fiascarelli A, Milano T, di Salvo ML, Nogués I, Pascarella S, Contestabile R. 2015. Molecular mechanism of PdxR – a transcriptional activator involved in the regulation of vitamin B6 biosynthesis in the probiotic bacterium Bacillus clausii. FEBS J 282:2966–2984. doi:10.1111/febs.13338 26059598

[62] Bonavita V. 1960. The reaction of pyridoxal 5-phosphate with cyanide and its analytical use. Arch Biochem Biophys 88:366–372. doi:10.1016/0003-9861(60)90252-6 13802445

[63] Naoi M, Ichinose H, Takahashi T, Nagatsu T. 1988. Sensitive assay for determination of pyridoxal-5-phosphate in enzymes using high-performance liquid chromatography after derivatization with cyanide. J Chromatogr 434:209–214. doi:10.1016/0378-4347(88)80077-x 3243815

[64] Kelley LA, Mezulis S, Yates CM, Wass MN, Sternberg MJE. 2015. The Phyre2 web portal for protein modeling, prediction and analysis. Nat Protoc 10:845–858. doi:10.1038/nprot.2015.053 25950237 PMC5298202

[65] Zdor RE. 2015. Bacterial cyanogenesis: impact on biotic interactions. J Appl Microbiol 118:267–274. doi:10.1111/jam.12697 25410133

[66] Siegień I, Bogatek R. 2006. Cyanide action in plants — from toxic to regulatory. Acta Physiol Plant 28:483–497. doi:10.1007/BF02706632

[67] Sehrawat A, Sindhu SS, Glick BR. 2022. Hydrogen cyanide production by soil bacteria: biological control of pests and promotion of plant growth in sustainable agriculture. Pedosphere 32:15–38. doi:10.1016/S1002-0160(21)60058-9

[68] Barclay M, Knowles CJ. 2001. Cyanide biodegradation by fungi, p 335–358. In Fungi in bioremediation. Cambridge University Press.

[69] Zlosnik JEA, Williams HD. 2004. Methods for assaying cyanide in bacterial culture supernatant. Lett Appl Microbiol 38:360–365. doi:10.1111/j.1472-765X.2004.01489.x 15059204

[70] Smith P, Cozart J, Lynn BK, Alberts E, Frangipani E, Schuster M. 2019. Bacterial cheaters evade punishment by cyanide. iScience 19:101–109. doi:10.1016/j.isci.2019.07.015 31357168 PMC6664145

[71] Yan H, Asfahl KL, Li N, Sun F, Xiao J, Shen D, Dandekar AA, Wang M. 2019. Conditional quorum-sensing induction of a cyanide-insensitive terminal oxidase stabilizes cooperating populations of Pseudomonas aeruginosa. Nat Commun 10:4999. doi:10.1038/s41467-019-13013-8 31676850 PMC6825135

[72] Nair C, Shoemark A, Chan M, Ollosson S, Dixon M, Hogg C, Alton E, Davies JC, Williams HD. 2014. Cyanide levels found in infected cystic fibrosis sputum inhibit airway ciliary function. Eur Respir J 44:1253–1261. doi:10.1183/09031936.00097014 25186256

[73] Gallagher LA, Manoil C. 2001. Pseudomonas aeruginosa PAO1 kills caenorhabditis elegans by cyanide poisoning. J Bacteriol 183:6207–6214. doi:10.1128/JB.183.21.6207-6214.2001 11591663 PMC100099

[74] Broderick KE, Chan A, Balasubramanian M, Feala J, Reed SL, Panda M, Sharma VS, Pilz RB, Bigby TD, Boss GR. 2008. Cyanide produced by human isolates of Pseudomonas aeruginosa contributes to lethality in Drosophila melanogaster. J Infect Dis 197:457–464. doi:10.1086/525282 18199034

[75] Randi EB, Zuhra K, Pecze L, Panagaki T, Szabo C. 2021. Physiological concentrations of cyanide stimulate mitochondrial complex IV and enhance cellular bioenergetics. Proc Natl Acad Sci USA 118:e2026245118. doi:10.1073/pnas.2026245118 33972444 PMC8157914

[76] Gotor C, García I, Aroca Á, Laureano-Marín AM, Arenas-Alfonseca L, Jurado-Flores A, Moreno I, Romero LC. 2019. Signaling by hydrogen sulfide and cyanide through post-translational modification. J Exp Bot 70:4251–4265. doi:10.1093/jxb/erz225 31087094

[77] Orillard E, Anaya S, Johnson MS, Watts KJ. 2021. Oxygen-induced conformational changes in the PAS-heme domain of the Pseudomonas aeruginosa Aer2 receptor. Biochemistry 60:2610–2622. doi:10.1021/acs.biochem.1c00452 34383467 PMC9148675

[78] Fukuyama K, Kunishima N, Amada F, Kubota T, Matsubara H. 1995. Crystal structures of cyanide- and triiodide-bound forms of arthromyces ramosus peroxidase at different pH values. Perturbations of active site residues and their implication in enzyme catalysis. J Biol Chem 270:21884–21892. doi:10.1074/jbc.270.37.21884 7665612

[79] Milani M, Ouellet Y, Ouellet H, Guertin M, Boffi A, Antonini G, Bocedi A, Mattu M, Bolognesi M, Ascenzi P. 2004. Cyanide binding to truncated hemoglobins: a crystallographic and kinetic study. Biochemistry 43:5213–5221. doi:10.1021/bi049870+ 15122887

[80] Mirajkar AL, Mittapelli LL, Nawale GN, Gore KR. 2018. Synthetic green fluorescent protein (GFP) chromophore analog for rapid, selective and sensitive detection of cyanide in water and in living cells. Sens Actuators B Chem 265:257–263. doi:10.1016/j.snb.2018.03.068

[81] Dai Z, Boon EM. 2010. Engineering of the heme pocket of an H-NOX domain for direct cyanide detection and quantification. J Am Chem Soc 132:11496–11503. doi:10.1021/ja101674z 20684546

[82] Mondal A, Chattopadhyay SK. 2022. Selective turn-on fluorescence sensing of cyanide using the pyridoxal platform of a Ni(II) complex. ACS Omega 7:40941–40949. doi:10.1021/acsomega.2c04063 36406569 PMC9670700

[83] Rahme LG, Stevens EJ, Wolfort SF, Shao J, Tompkins RG, Ausubel FM. 1995. Common virulence factors for bacterial pathogenicity in plants and animals. Science 268:1899–1902. doi:10.1126/science.7604262 7604262

[84] Bertani G. 2004. Lysogeny at mid-twentieth century: P1, P2, and other experimental systems. J Bacteriol 186:595–600. doi:10.1128/JB.186.3.595-600.2004 14729683 PMC321500

[85] Hoang TT, Karkhoff-Schweizer RR, Kutchma AJ, Schweizer HP. 1998. A broad-host-range FLP-FRT recombination system for site-specific excision of chromosomally-located DNA sequences: application for isolation of unmarked Pseudomonas aeruginosa mutants. Gene 212:77–86. doi:10.1016/s0378-1119(98)00130-9 9661666

[86] Shanks RMQ, Caiazza NC, Hinsa SM, Toutain CM, O’Toole GA. 2006. Saccharomyces cerevisiae-based molecular tool kit for manipulation of genes from gram-negative bacteria. Appl Environ Microbiol 72:5027–5036. doi:10.1128/AEM.00682-06 16820502 PMC1489352

[87] Cornell WC, Morgan CJ, Koyama L, Sakhtah H, Mansfield JH, Dietrich LEP. 2018. Paraffin embedding and thin sectioning of microbial colony biofilms for microscopic analysis. J Vis Exp:57196. doi:10.3791/57196 29630036 PMC5933251

[88] Sakhtah H, Koyama L, Zhang Y, Morales DK, Fields BL, Price-Whelan A, Hogan DA, Shepard K, Dietrich LEP. 2016. The Pseudomonas aeruginosa efflux pump MexGHI-OpmD transports a natural phenazine that controls gene expression and biofilm development. Proc Natl Acad Sci USA 113. doi:10.1073/pnas.1600424113 PMC4922186 27274079

[89] Dietrich LEP, Price-Whelan A, Petersen A, Whiteley M, Newman DK. 2006. The phenazine pyocyanin is a terminal signalling factor in the quorum sensing network of Pseudomonas aeruginosa. Mol Microbiol 61:1308–1321. doi:10.1111/j.1365-2958.2006.05306.x 16879411

[90] Rappsilber J, Mann M, Ishihama Y. 2007. Protocol for micro-purification, enrichment, pre-fractionation and storage of peptides for proteomics using stagetips. Nat Protoc 2:1896–1906. doi:10.1038/nprot.2007.261 17703201

