# Supplementary material for: MpaR-driven expression of an orphan terminal oxidase subunit supports Pseudomonas aeruginosa biofilm respiration and development during cyanogenesis
Source: mBio. 2023 Dec 19;15(1):e02926-23. doi: 10.1128/mbio.02926-23 (PMC10790758; doi:10.1128/mbio.02926-23)
Supplement: File S1 — Accompaniment to Figure 7. [file mbio.02926-23-s0001.pdf]

Supplementary File 1

We used both the DALI server (1) and the PHYRE2 Protein Fold Recognition Server (2) to search for structural homologs of the Alpha-Fold (AF) (3, 4) model of MpaR against all available 3D structures in the Protein Data Bank (PDB). DALI indicated that the C-terminal domain of MpaR is a PLP-dependent transferase. The top 15 structural homologs appear to function as α-aminoadipate aminotransferases. PHYRE2 also built models for the PLP-binding domain based on structures that are available in PDB. However, out of 20 models produced for MpaR, PHYRE2 built 18 for the C-terminal domain and two (#12 and #13) for the full protein using two-domain structures of *Bacillus clausii* PdxR (PDB code: 7ZLA) and *Bacillus subtilis* GabR (PDB code: 4N0B) (5). Both PdxR and GabR bind PLP and form homodimers with swapped DNA-binding domains in each protomer, suggesting that binding of PLP likely triggers the domain swapping in these proteins and potentially also in MpaR.

To explore this possibility, we first built a model of the MpaR homodimer. Because the AF model of MpaR has the N-terminal DNA-binding domain in a closed conformation, we used AlphaFold2-multimer to generate models of the MpaR homodimer. Surprisingly, none of the five models indicated domain swapping between the two protomers of MpaR (Figure 1). This suggests that domain swapping may occur upon PLP binding and/or that addition of cyanide to PLP, which results in the formation of a cyanohydrin derivative (PLP-CN), makes MpaR (more) amenable to DNA binding.

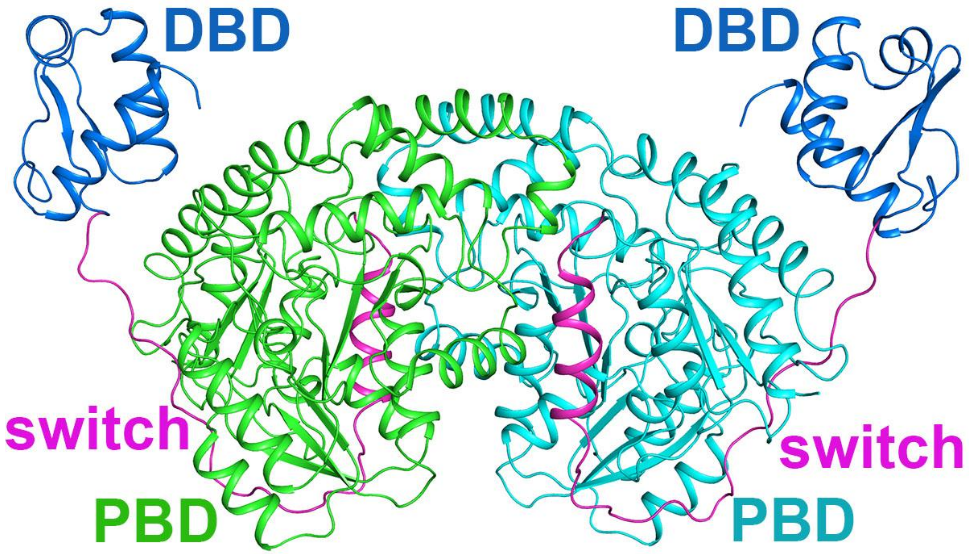

Figure 1. An AF2-multimer model of the MpaR homodimer (green and cyan). PDB (green/cyan) and DBD (marine), denote PLP-binding and DNA-binding domains, respectively. The switch sequences that connect the two domains are shown in magenta. This figure and subsequent figures were generated using PyMOL.

In fact, overlay of two structures of GabR bound to imidazole (PDB code: 4MGR) alone and the derivatized PLP product (PDB code: 4N0B) suggests that the aromatic pyridine group of PLP is most likely the driving force to induce the conformational change that results in domain swapping, as imidazole alone, mimicking the PLP pyridine group, does induce domain swapping (Figure 2). There is an abundance of aromatic and methionine residues in the active site of GabR, which collectively form a network of π-π and S-π interactions with the substrate/product

(Figure 2). The PLP modification could thus cause a subtle conformational change in the side chains of these aromatic and methionine residues, which in turn could result in optimal positioning of the DNA-binding domain of GabR. Similarly, the active site of each subunit of the PdxR dimer in complex with modified PLP and DNA is enriched with aromatic residues, which likely play a similar role. Taken together, these crystal structures provide snapshots as to how PLP and its modified form induce domain swapping and enhance the DNA binding capabilities of GabR, PdxR, and MpaR.

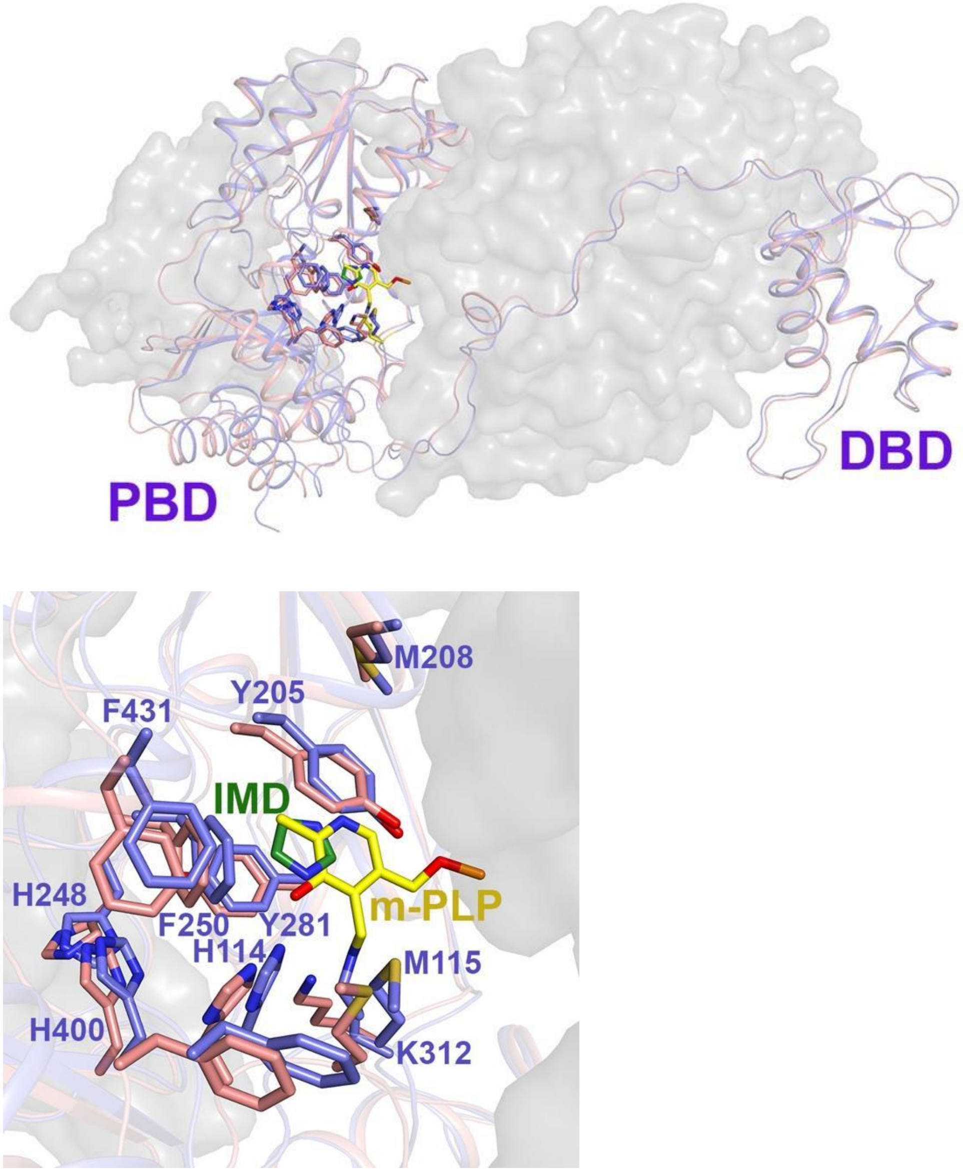

Figure 2. Structural overlay of two GabR dimers bound to imidazole and modified PLP. Top: For the sake of clarity, only subunit A (salmon) of the GabR dimer (PDB code: 4MGR) bound to imidazole (dark green) is shown together with the GabR dimer (purple for subunit A and grey surface for subunit B) bound to modified PLP (PDB code: 4N0B). Bottom: Close-up view of the active site of two GabR dimers. Important aromatic/methionine residues and K312 are depicted as stick models and labeled. The active site of GabR is enriched with aromatic residues.

The overlay of the AF model of apo MpaR with that generated by PHYRE2 reveals an intriguing result (Figure 3). The AF model of MpaR shows the N-terminal DNA-binding domain (magenta) of the protein is adjacent to the PLP-dependent domain (yellow) and away from the dimer interface, thus it is not involved in dimerization. More importantly, an α-helix (labeled in magenta) in the AF model of apo MpaR becomes a loop in the presumably, activated, PLP-bound MpaR dimer. There is ample evidence for such a conversion in which a segment of a protein containing proline residues in abundance plays a critical role (6). As shown in Figure 3, MpaR contains a proline-rich region spanning from residue 105 to123. Four prolines (P105, P109, P115, P117) are clustered near F114 and Y284, the side chain of both face the PLP-binding pocket (discussed further below). We therefore hypothesize that binding of PLP, and particularly PLP-CN, will trigger a significant conformational change in the DNA-binding domain such that MpaR becomes amenable to efficient DNA binding.

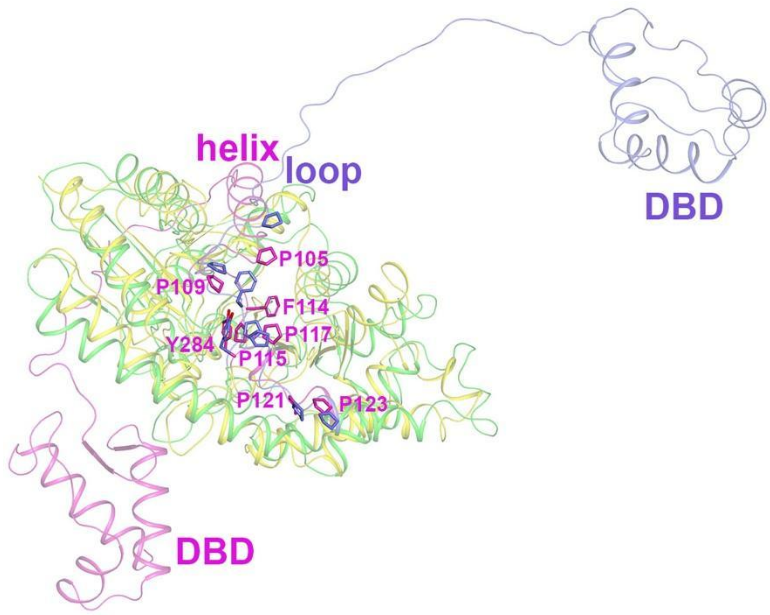

Figure 3. Overlay of two models of MpaR generated by Alpha-Fold (yellow/magenta) and PHYRE2 (green/purple). The side chains of important residues that may contribute to a drastic conformational change of MpaR are shown as labeled stick models.

To further our understanding of the mode of binding of PLP and PLP-CN, we performed in situ Induced-Fit docking of each substrate using Maestro (Schrödinger Release 2023-3: Maestro, Schrödinger, LLC, New York, NY, 2023). The result of each docking together with the associated docking score are shown in Figures 4&5. We modeled PLP as a non-covalent substrate, because the cyanohydrin moiety can only be generated when CN^- attacks the carbonyl group of either an aldehyde or a ketone (7). Accordingly, covalent modification of K314 could remove the carbonyl moiety of PLP aldehyde. We therefore hypothesize that PLP and K314 of MpaR would not form a covalent adduct. The docking data suggest that the cyanohydrin derivative of PLP (PLP-CN) binds stronger to MpaR than to PLP. In the absence of any structure data for MpaR in complex with either PLP or PLP-CN and DNA, we can only speculate that there are three residues that along with the proline-rich region collectively are responsible for the conformational change in MpaR. L179 resides at the end of the a-helix, which is a well-known phosphate binding loop (P-loop) (Figure 6). L179 forms hydrophobic interactions with F203, while both residues interact with the aromatic ring of each substrate in their respective

Induced-Fit model. However, a subtle shift in the side chain of F203 is visible when PLP gets modified to PLP-CN. Consequently, F203 forms stronger π-π interactions with the aromatic ring of PLP-CN as opposed to those in PLP. It is therefore conceivable that stronger interactions between F203 and PLP-CN could cause a conformational change in M248, Y284, and F114, the last residue of which is in the proximity of the proline-rich region, thereby inducing a cascade of conformational changes in the DNA-binding domain. Lastly, we made a model of the entire complex of MpaR dimer bound to PLP-CN and DNA (Figure 7).

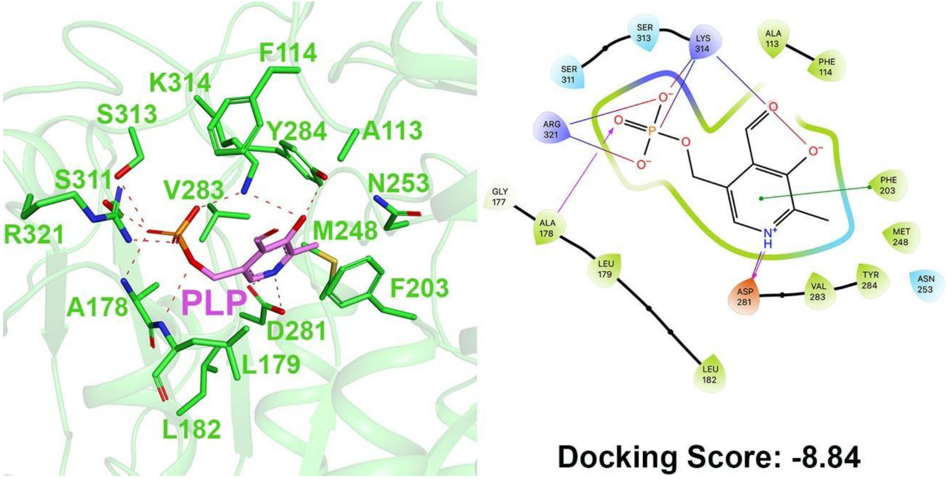

Figure 4. Induced-Fit docking of PLP into the AF model of MpaR. Left: All interacting residues (green) and PLP (violet) are shown as stick models and labeled. Red dashed lines depict hydrogen bonds. Right: Schematic representation of interactions of PLP (black) with surrounding residues of MpaR. The docking Score suggests strong interactions.

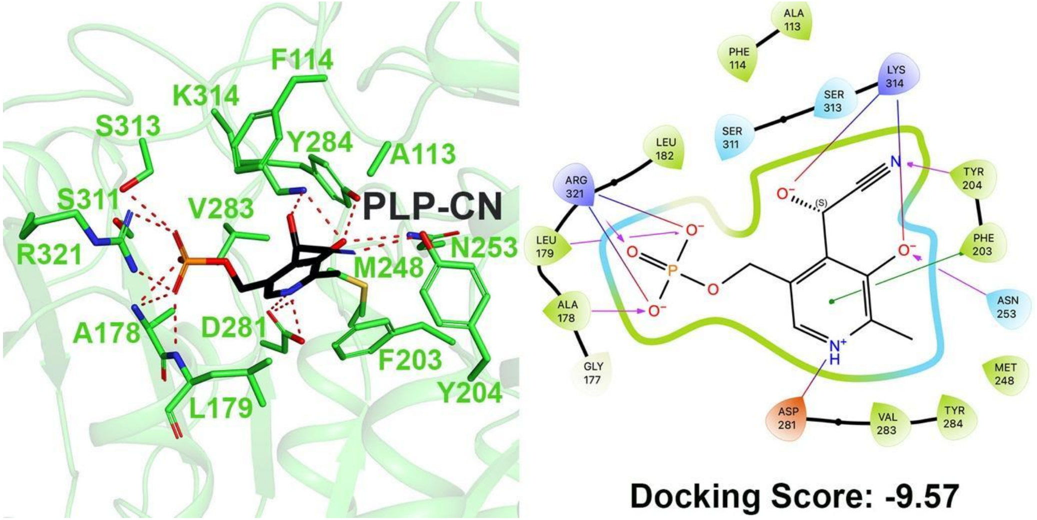

Figure 5. Induced-Fit docking of PLP-CN into the AF model of MpaR. Left: All interacting residues (green) and PLP-CN (black) are shown as stick models and labeled. Red dashed lines depict hydrogen bonds. Right: Schematic representation of interactions of PLP-CN (black) with surrounding residues of MpaR. The docking Score suggests stronger interactions as compared with those of PLP.

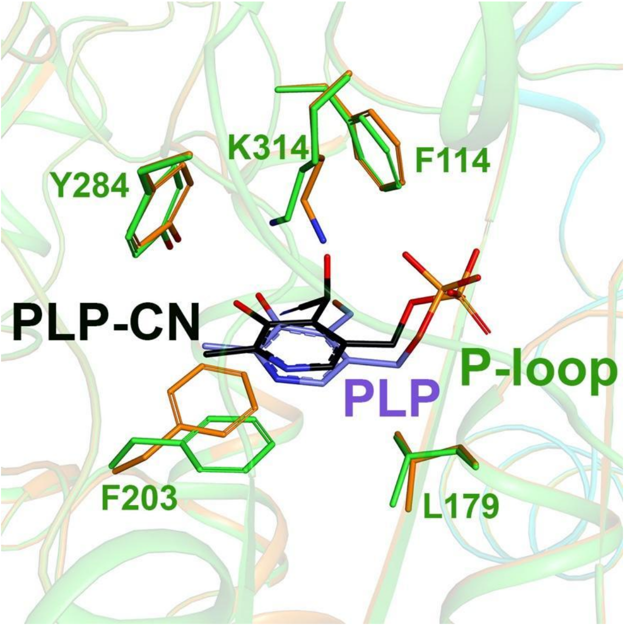

Figure 6. Overlay of the Induced-Fit models of MpaR in complex with PLP and PLP-CN. Cartoon representation of MpaR (orange) with PLP (purple) and MpaR (green) with PLP-CN (black). Four hydrophobic residues (L179, F203, F114, Y284) together with K314 are shown as stick models and labeled. The phosphate binding loop (P-loop) is also labeled.

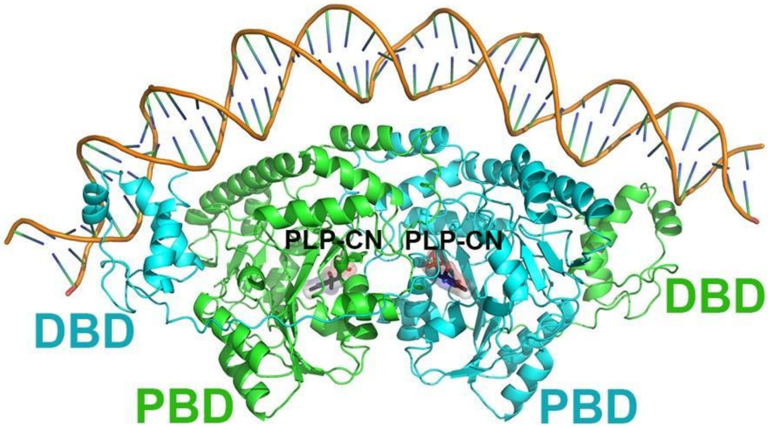

Figure 7. A model of the MpaR homodimer (green and cyan) bound to PLP-CN (black for carbon atoms) and double stranded DNA (orange/blue/green). PBD and DBD denote PLP-binding and DNA-binding domains, respectively. DNA from the cryoEM structure of PdxR (PDB code: 7ZLA) was used for docking.

Method

Induced-Fit docking of PLP and PLP-CN

Induced-Fit docking of each ligand was performed using Maestro (Schrödinger Suite). Both PLP and the cyanohydrin derivative of PLP (PLP-CN) were first prepared for in silico docking in the OPLS4 force field by the LigPrep program of Maestro. Ionization states at pH 7.0 ± 2.0 were set at default values and all potential tautomers (isomers) were generated. Maestro produced 4 and 12 tautomers for PLP and PLP-CN, respectively. The Alpha-Fold (AF) model of MpaR was used as the receptor for both dockings with Lys-314 as the most important site. Protein preparation wizard of Maestro was applied to preprocess and refine the AF model of MpaR with addition of hydrogen atoms in the OPLS4 force filed before generation of the receptor grid. The Induced-Fit docking was performed by the Glide program implemented in Maestro. AlphaFold2-multimer (8) was used for generation of 5 models of the MpaR dimer. Double stranded DNA from crystal structure of PdxR (PDB code: 7ZLA) was docked on the PdxR-based model of MpaR generated by PHYRE2. The entire complex was then subjected to geometric optimization and minimization using the Prime program in Schrödinger Suite.

References

1. Holm L, Laakso LM. 2016. Dali server update. Nucleic Acids Res 44:W351–5.
2. Kelley LA, Mezulis S, Yates CM, Wass MN, Sternberg MJE. 2015. The Phyre2 web portal for protein modeling, prediction and analysis. Nat Protoc 10:845–858.
3. Varadi M, Anyango S, Deshpande M, Nair S, Natassia C, Yordanova G, Yuan D, Stroe O, Wood G, Laydon A, Žídek A, Green T, Tunyasuvunakool K, Petersen S, Jumper J, Clancy E, Green R, Vora A, Lutfi M, Figurnov M, Cowie A, Hobbs N, Kohli P, Kleywegt G, Birney E, Hassabis D, Velankar S. 2022. AlphaFold Protein Structure Database: massively expanding the structural coverage of protein-sequence space with high-accuracy models. Nucleic Acids Res 50:D439–D444.
4. Jumper J, Evans R, Pritzel A, Green T, Figurnov M, Ronneberger O, Tunyasuvunakool K, Bates R, Žídek A, Potapenko A, Bridgland A, Meyer C, Kohl SAA, Ballard AJ, Cowie A, Romera-Paredes B, Nikolov S, Jain R, Adler J, Back T, Petersen S, Reiman D, Clancy E, Zielinski M, Steinegger M, Pacholska M, Berghammer T, Bodenstein S, Silver D, Vinyals O, Senior AW, Kavukcuoglu K, Kohli P, Hassabis D. 2021. Highly accurate protein structure prediction with AlphaFold. Nature 596:583–589.

5. Edayathumangalam R, Wu R, Garcia R, Wang Y, Wang W, Kreinbring CA, Bach A, Liao J, Stone TA, Terwilliger TC, Hoang QQ, Belitsky BR, Petsko GA, Ringe D, Liu D. 2013. Crystal structure of Bacillus subtilis GabR, an autorepressor and transcriptional activator of gabT. Proc Natl Acad Sci U S A 110:17820–17825.

6. Alcantara J, Stix R, Huang K, Connor A, East R, Jaramillo-Martinez V, Stollar EJ, Ball KA. 2021. An Unbound Proline-Rich Signaling Peptide Frequently Samples Cis Conformations in Gaussian Accelerated Molecular Dynamics Simulations. Front Mol Biosci 8:734169.

7. Zuhra K, Szabo C. 2022. The two faces of cyanide: an environmental toxin and a potential novel mammalian gasotransmitter. FEBS J 289:2481–2515.

8. Bryant P, Pozzati G, Elofsson A. 2022. Improved prediction of protein-protein interactions using AlphaFold2. Nat Commun 13:1265.
