# Supplementary material for: MpaR-driven expression of an orphan terminal oxidase subunit supports Pseudomonas aeruginosa biofilm respiration and development during cyanogenesis
Source: mBio. 2023 Dec 19;15(1):e02926-23. doi: 10.1128/mbio.02926-23 (PMC10790758; doi:10.1128/mbio.02926-23)
Supplement: Supplemental Figures and Tables — Figures 1-8 and Tables 1-3. [file mbio.02926-23-s0002.pdf]

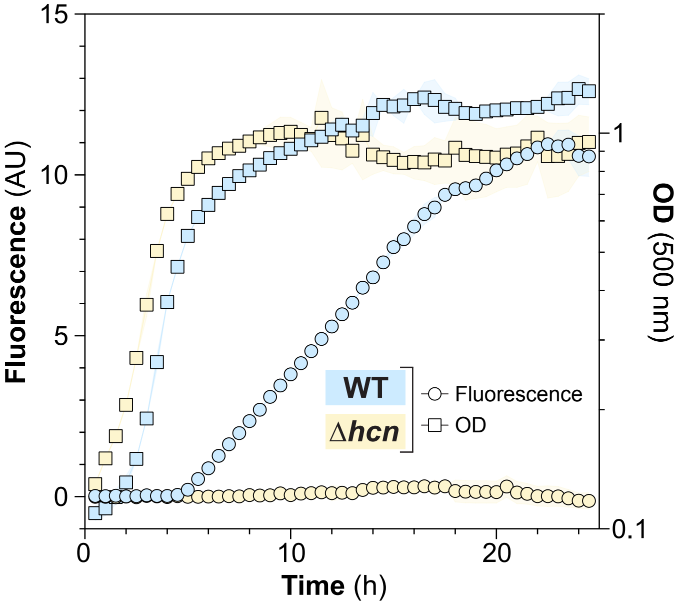

Figure S1. Endogenous cyanide enhances P*ccoN4-gfp* activity. Activity of *ccoN4* promoter during growth in liquid culture (1% tryptone) reported as GFP fluorescence. Data represents the average of three biological replicates with shading indicating standard deviation. Arrow indicates the onset of stationary phase.

Smiley, Sekaran et al.
SI Figure 2

A

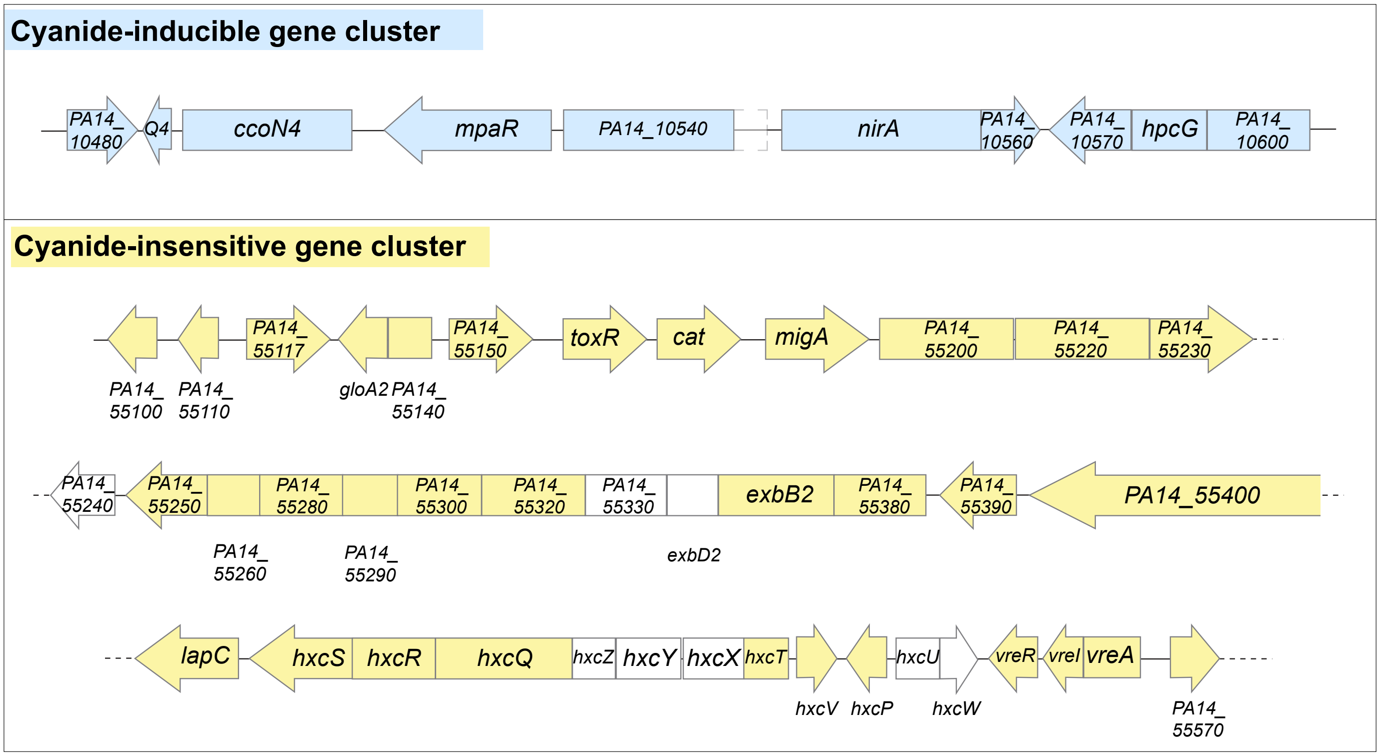

B

Gene ID	Gene ID (PA14)	Name	Fold change	P-value	Function
**PA4132**	PA14_10530	mpaR	-421.429	2.411E-159	hypothetical protein
**PA0713**	PA14_55110		-331.686	4.013E-112	hypothetical protein
**PA0690**	PA14_55400		-107.683	1.07E-87	hypothetical protein
**PA4133**	PA14_10500	ccoN4	-98.291	0E+00	cytochrome c oxidase subunit (cbb3-type)
**PA4130**	PA14_10550	nirA	-68.082	0E+00	probable sulfite or nitrite reductase
**PA4129**	PA14_10560		-67.126	1.296E-87	hypothetical protein
**PA0689**	-	lapB	-65.337	4.851E-53	low-molecular-weight alkaline phosphatase B
**PA0705**	PA14_55180	migA	-60.714	9.662E-55	alpha-1,6-rhamnosyltransferase MigA
**PA0714**	PA14_55100		-59.844	2.485E-46	hypothetical protein
**PA0704**	PA14_55200		-56.050	2.459E-47	probable amidase
**PA0706**	PA14_55170	cat	-43.080	4.794E-42	chloramphenicol acetyltransferase
**PA4128**	PA14_10570		-41.140	7.095E-58	conserved hypothetical protein
**PA0708**	PA14_55150		-32.109	9.782E-32	probable transcriptional regulator
**PA0709**	PA14_55140		-30.483	1.099E-29	hypothetical protein
**PA4131**	PA14_10540		-25.616	0E+00	probable iron-sulfur protein
**PA0688**	PA14_55410	lapC	-24.192	2.614E-26	probable binding protein component of ABC transporter
**PA0703**	PA14_55220		-13.842	1.126E-15	probable major facilitator superfamily (MFS) transporter
**PA4134**	PA14_10490	ccoQ4	-11.920	4.72E-92	hypothetical protein
**PA0710**	PA14_55130	gloA2	-11.668	5.796E-12	lactoylglutathione lyase
**PA0696**	PA14_55320		-11.547	3.226E-13	hypothetical protein
**PA0673**	PA14_55570		-11.112	1.227E-12	hypothetical protein
**PA0685**	PA14_55450	hxcQ	-10.163	1.709E-11	HxcQ probable type II secretion system protein
**PA0701a**	PA14_55250		-9.550	6.089E-11	hypothetical protein
**PA3022**	PA14_24980		-8.310	1.264E-143	hypothetical protein
**PA0712**	PA14_55117a		-7.831	4.937E-09	hypothetical protein
**PA0679**	PA14_55510	hxcP	-7.714	1.008E-07	hypothetical protein
**PA0692**	PA14_55380		-7.513	5.386E-08	hypothetical protein
**PA0711**	PA14_55117b		-7.429	1.696E-07	hypothetical protein
**PA0702**	PA14_55230		-7.426	2.172E-08	hypothetical protein
**PA0676**	PA14_55540	vreR	-7.166	4.816E-08	sigma factor regulator, VreR
**PA4127**	PA14_10590	hpcG	-7.096	4.831E-24	2-oxo-hept-3-ene-1,7-dioate hydratase
**PA0693**	PA14_55360	exbB2	-6.749	2.464E-07	transport protein ExbB2
**PA0701**	PA14_55250		-6.688	3.966E-07	probable transcriptional regulator
**PA0699**	PA14_55280		-6.082	9.401E-06	probable peptidyl-prolyl cis-trans isomerase, PpiC-type
**PA0681**	PA14_55490	hxcT	-6.035	5.105E-06	HxcT pseudopilin
**PA0707**	PA14_55160	toxR	-4.973	4.048E-05	transcriptional regulator ToxR
**PA0674**	PA14_55560	vreA	-4.859	1.634E-04	transcriptional regulators VreA
**PA0686**	PA14_55440	hxcR	-4.453	4.338E-04	HxcR probable type II secretion system protein
**PA2331**	PA14_34460		-4.283	7.562E-50	hypothetical protein
**PA0698**	PA14_55290		-4.164	1.426E-03	hypothetical protein
**PA3912**	PA14_13300		-4.147	8.174E-13	hypothetical protein
**PA0687**	PA14_55430	hxcS	-3.900	2.921E-03	HxcS probable type II secretion system protein
**PA0675**	PA14_55550	vreI	-3.776	2.418E-03	ECF sigma factor, VreI
**PA0691**	PA14_55390		-3.714	4.886E-03	hypothetical protein
**PA4135**	PA14_10480		-3.663	7.044E-23	probable transcriptional regulator
**PA4126**	PA14_10600		-3.531	2.242E-11	probable major facilitator superfamily (MFS) transporter
**PA0697**	PA14_55300		-3.450	7.881E-03	hypothetical protein
**PA0680**	PA14_55500	hxcV	-3.364	1.139E-02	HxcV putative pseudopilin
**PA0700**	PA14_55260		-3.308	1.226E-02	hypothetical protein
**PA1913**	PA14_39790		-3.260	4.169E-20	hypothetical protein

Figure S2. MpaR and cyanide affect the expression of a gene cluster in *P. aeruginosa*. (**A**) A previously published RNAseq study compared gene expression in PAO1 Δ*mpaR* to WT (see also panel B) and found two chromosomal regions with decreased gene expression (1). Expression of genes in one of these clusters (highlighted in blue) is induced by cyanide (2). Genes in the other cluster that were only affected by *mpaR* but not cyanide are indicated in yellow. (**B**) Results of the RNAseq analysis described in Wang et al. (1), which identified genes differentially regulated between PAO1 Δ*mpaR* and WT. Genes highlighted in blue have also been shown to be induced by cyanide. Genes in yellow are found in the second cluster in panel (A) and their expression was not affected by cyanide (2).

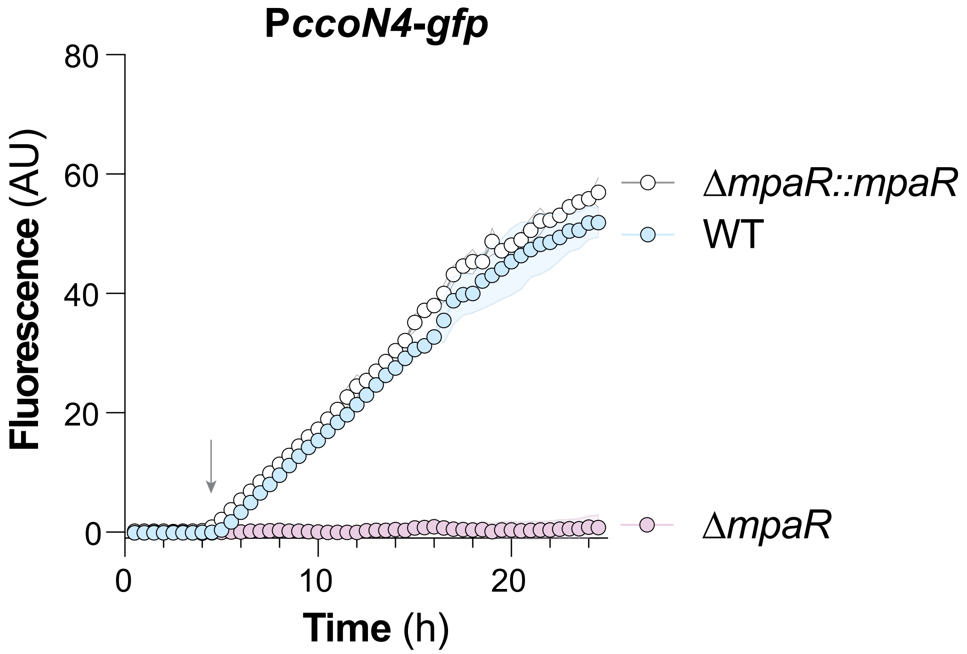

Figure S3. Complementation of *mpaR* restores expression of *ccoN4*. Activities of the *ccoN4* promoter during growth in liquid culture (1% tryptone) reported as GFP fluorescence. Data represent the mean of three biological replicates with shading indicating standard deviation. Arrow indicates the onset of stationary phase.

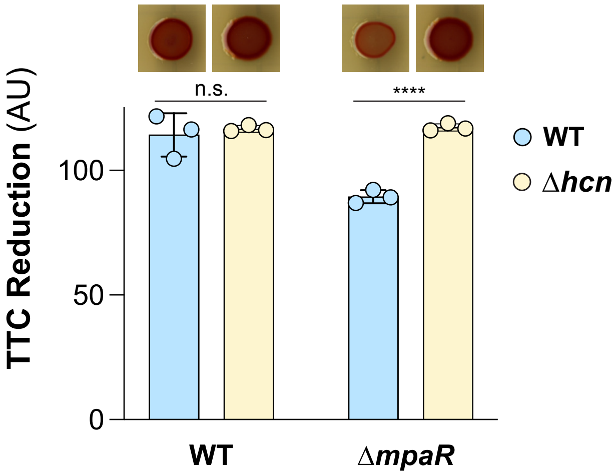

Figure S4. MpaR is required for full TTC reduction in the presence of cyanide. TTC reduction (red coloration) by biofilms of the indicated strains, quantified as the average saturation in the red hue for all pixels in the colony area. Data points represent biological replicates and error bars indicate standard deviation. P-values were calculated using unpaired, two-tailed t tests (****p<0.0001).

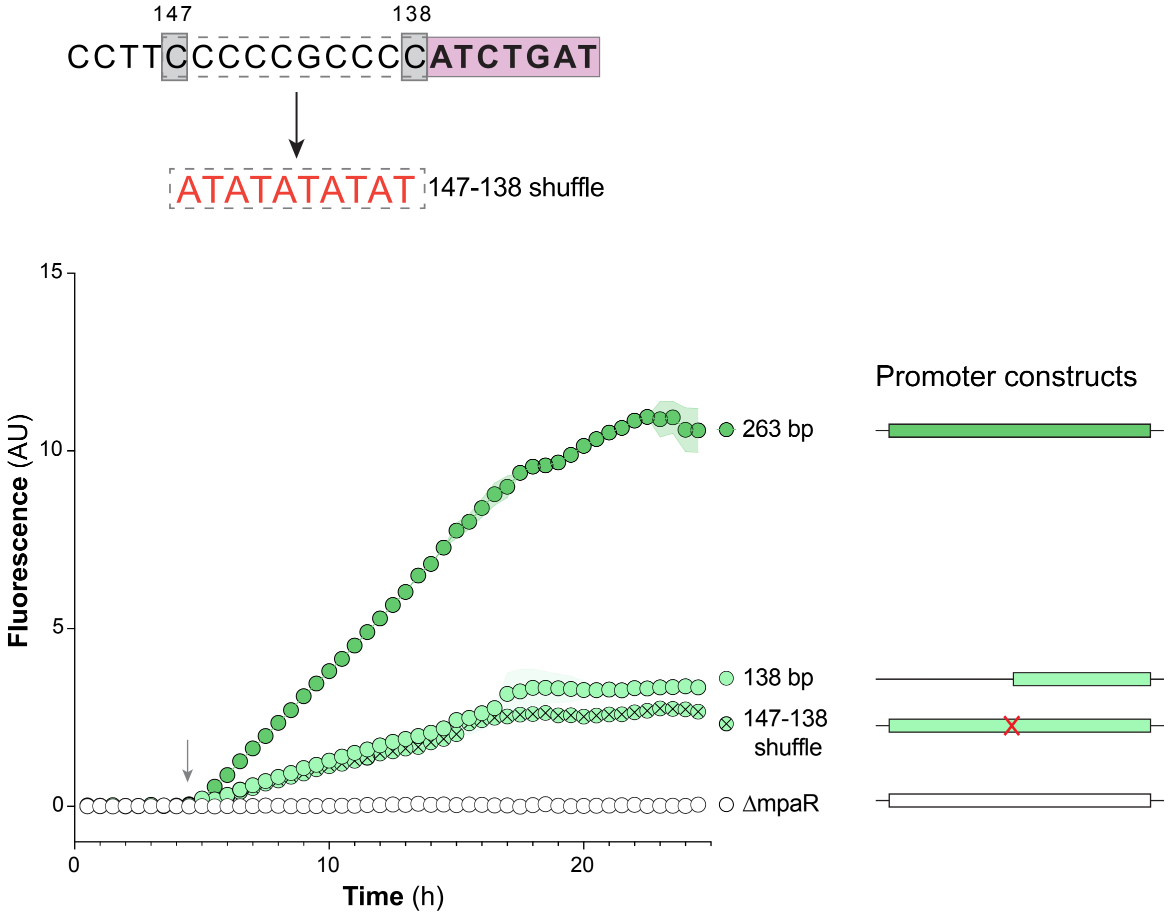

Figure S5. A GC-rich region upstream of the palindromic motif is required for full expression of the *ccoN4* promoter. The palindromic motif that is required for *ccoN4* expression (**Figure 4**) is indicated as an orange rectangle. The ten base pairs immediately preceding this motif (147-138) were mutated from CCCCCGCCCC to ATATATATAT in a reporter containing the full intergenic region and the strain is denoted as "147-138 shuffle". A strain containing the full intergenic region in the Δ*mpaR* background is shown as a negative control. Values shown represent the average of three biological replicates and shading indicates standard deviation. Arrow indicates the onset of stationary phase.

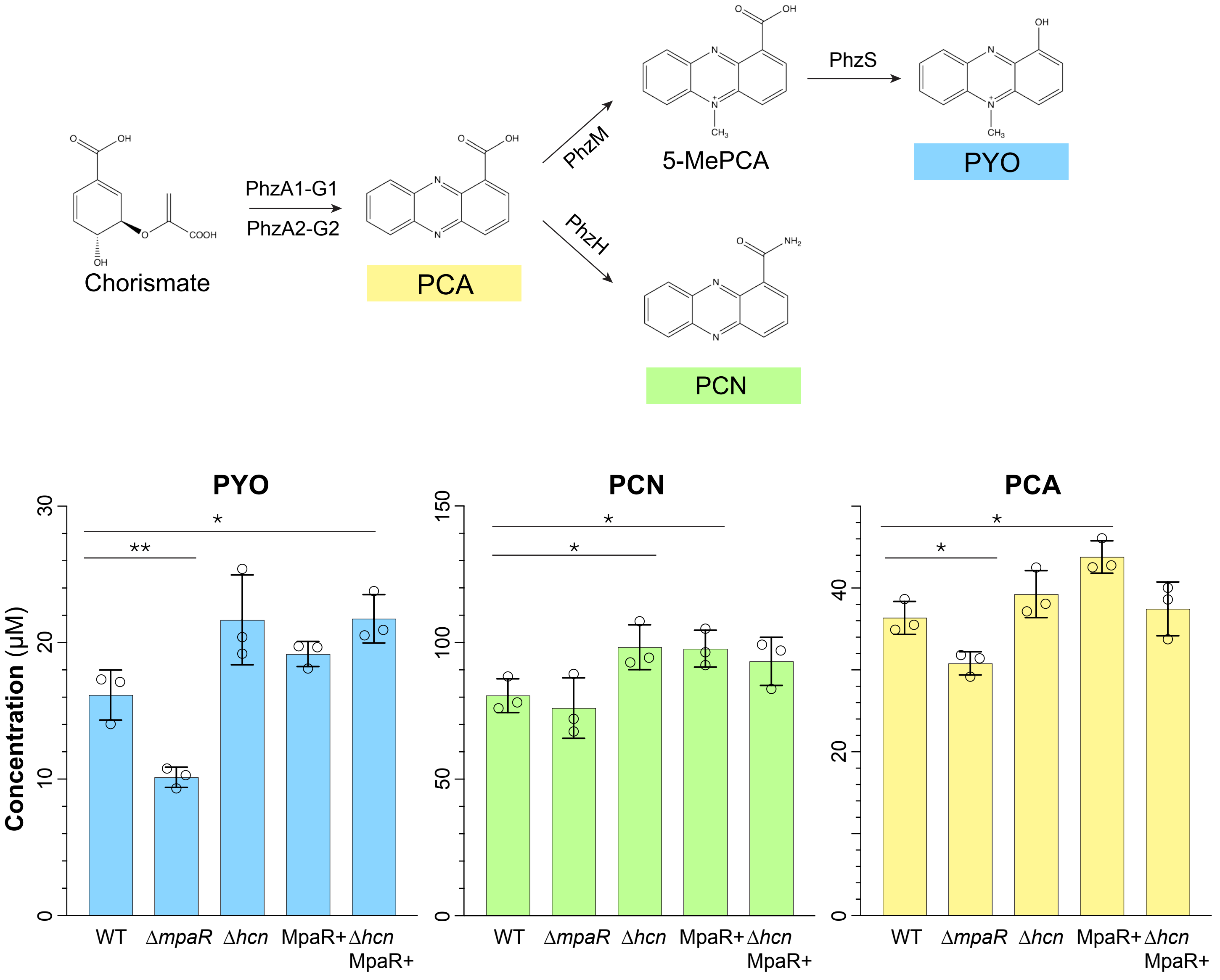

Figure S6. Phenazine quantification indicates a modest role for MpaR in the regulation of phenazine production. (**A**) Schematic of the phenazine biosynthetic pathway whereby chorismate is converted to PCA followed by differentiation into derivatives including PCN and PYO. (**B**) High performance liquid chromatography quantification of phenazines produced by biofilms grown for 72 hours on 1% tryptone 1% agar. P-values were calculated using unpaired, two-tailed t tests (*$p<0.05$; **$p<0.01$).

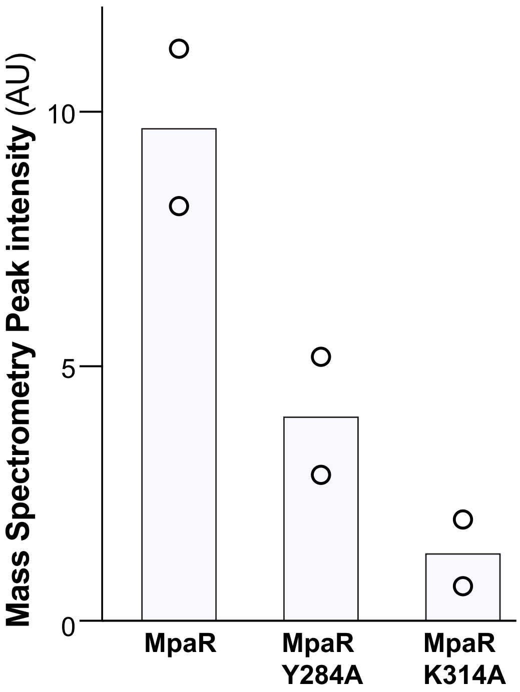

Figure S7. MpaR point mutants show decreased abundance, consistent with MpaR-dependent control of *PA14_10540-mpaR* expression. Peak intensities of MpaR WT and each of the indicated point mutants detected, by mass spectrometry analysis, in extracts from three-day-old biofilms formed by the corresponding strains. Each data point represents a biological replicate. The effects of these mutations on *PA14_10540-mpaR* expression was assessed by microscopic analysis as shown in Figure 7D.

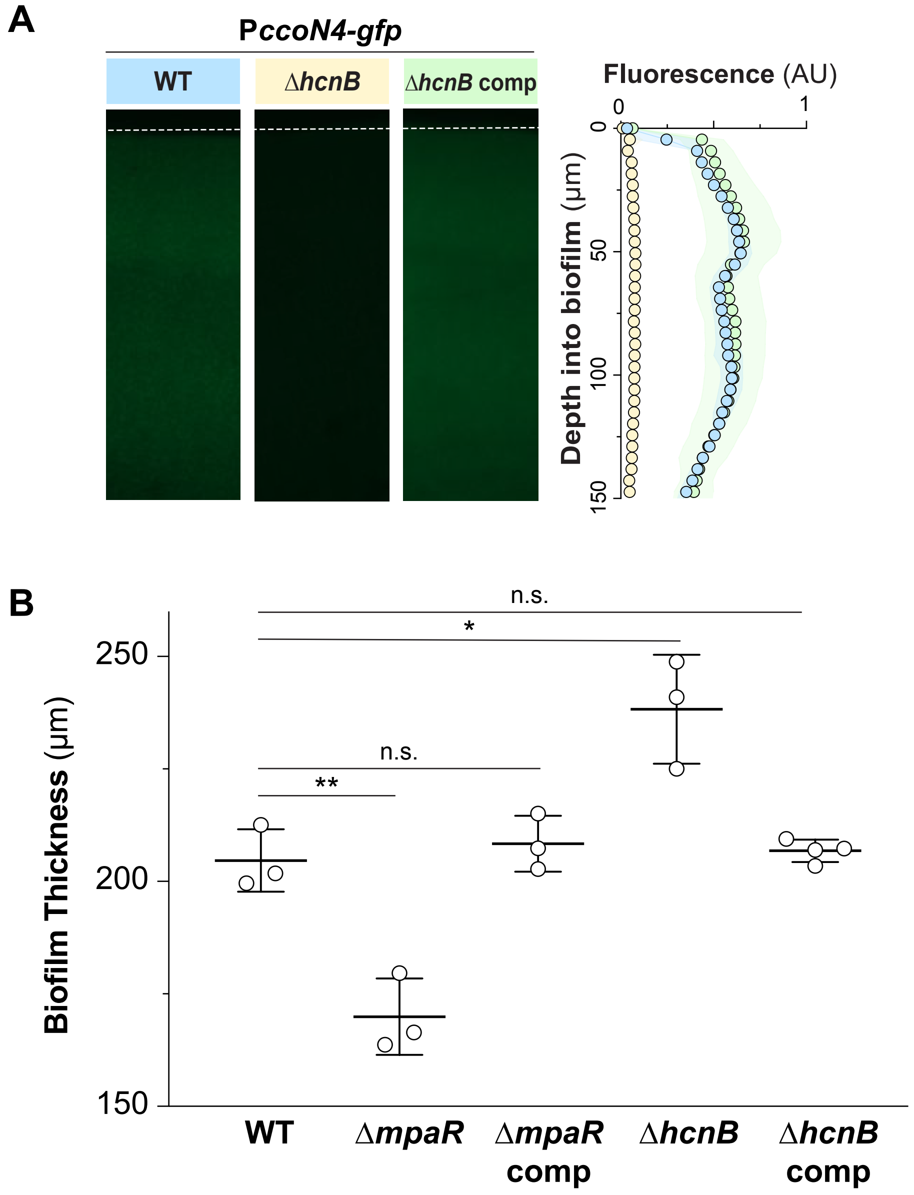

Figure S8. Complementation of the Δ*hcnB* and Δ*mpaR* deletion strains restores WT phenotypes. (**A**) Representative thin-section images of the indicated strains, each containing the P*ccoN4-gfp* reporter. Biofilms were grown for three days before harvesting and preparation of thin sections. For quantification, shown on the right, experiments were performed with biological triplicates and shading indicates standard deviation. (**B**) Thicknesses of biofilms formed by the indicated strains. Biofilms were grown for three days before harvesting and preparation of thin sections. Data points represent biological replicates and error bars represent standard deviation. P-values were calculated using unpaired, two-tailed t tests (*p<0.01, **p<0.001).

Table S1. Bacterial strains used in this study.

Number	Strain	Description	Source
Pseudomonas aeruginosa strains			
LD0	UCBPP-PA14 (WT)	Clinical isolate UCBPP-PA14.	(3)
LD2827	Δ*hcn*	PA14 with the *hcnA-C* (*PA14_36310-PA14_36330*) operon deleted.	(4)
LD2833	Δ*ccoN4*	PA14 with *ccoN4* (*PA14_10500*) deleted.	(4)
LD2829	Δ*hcn*Δ*ccoN4*	PA14 with the *hcnA-C* (*PA14_36310-PA14_36330*) operon and *ccoN4* (*PA14_10500*) deleted.	(4)
LD1888	Δ*ccoN1*Δ*ccoN2*	PA14 with *ccoN1* (*PA14_44370*) and *ccoN2* (*PA14_44340*) deleted.	(4)
LD2830	Δ*hcn*Δ*ccoN1*Δ*ccoN2*	PA14 with the *hcnA-C* (*PA14_36310- PA14_36330*) operon, *ccoN1* (*PA14_44370*) and *ccoN2* (*PA14_44340*) deleted.	(4)
LD1977	Δ*ccoN1*Δ*ccoN2*Δ*ccoN4*	PA14 with *ccoN1* (*PA14_44370*), *ccoN2* (*PA14_44340*), and *ccoN4* (*PA14_10500*) deleted.	(4)
LD2831	Δ*hcn*Δ*ccoN1*Δ*ccoN2* Δ*ccoN4*	PA14 with the *hcnA-C* (*PA14_36310-PA14_36330*) operon, *ccoN1* (*PA14_44370*), *ccoN2* (*PA14_44340*), and *ccoN4* (*PA14_10500*) deleted.	(4)
LD915	Δ*anr*	PA14 with *anr* (*PA14_44490*) deleted.	(5)
LD3884	Δ*mpaR*	PA14 with *mpaR* (*PA14_10530*) deleted. Made by mating pLD3774 into PA14.	This study
LD4867	Δ*hcn*Δ*mpaR*	PA14 with the *hcnA-C* (*PA14_36310-PA14_36330*) operon and *mpaR* (*PA14_10530*) deleted. Made by mating pLD3774 into LD2827.	This study
LD4317	Δ*mpaR::mpaR*	PA14 Δ*mpaR* (*PA14_10530*) strain with wild-type *mpaR* complemented back into the site of deletion. Made by mating pLD4286 into LD3884.	This study
LD2822	Δ*hcnB*	PA14 with *hcnB* (*PA14_36320*) deleted. Made by mating pLD2790 into PA14.	This study
LD5159	Δ*hcnB::hcnB*	PA14 Δ*hcnB* (*PA14_36320*) strain with wild-type *hcnB* complemented back into the site of deletion. Made by mating pLD5158 into LD2822.	This study
LD2784	PA14 *attB::*P*ccoN1-gfp*	PA14 containing a construct in the *attB* site that expresses *gfp* under control of the 500 bp region upstream of *ccoN1* (*PA14_44340*).	(4)
LD2786	PA14 *attB::*P*ccoN2-gfp*	PA14 containing a construct in the *attB* site that expresses *gfp* under control of the 500 bp region	(4)

		upstream of *ccoN2* (*PA14_44370*).	
LD4277	PA14 *attB::*P*ccoN4-gfp*	PA14 containing a construct in the *attB* site that expresses *gfp* under control of the 263 bp region upstream of *ccoN4* (*PA14_10500*). Made by mating pLD4258 into PA14.	This study
LD4083	Δ*anr attB::*P*ccoN1-gfp*	Δ*anr* containing a construct in the *attB* site that expresses *gfp* under control of the 500 bp region upstream of *ccoN1*. Made by mating pLD2766 into LD915.	This study
LD4084	Δ*anr attB::*P*ccoN2-gfp*	Δ*anr* containing a construct in the *attB* site that expresses *gfp* under control of the 500 bp region upstream of *ccoN2*. Made by mating pLD2767 into LD915.	This study
LD4342	Δ*anr attB::*P*ccoN4-gfp*	Δ*anr* containing a construct in the *attB* site that expresses *gfp* under control of the 263 bp region upstream of *ccoN4*. Made by mating pLD4258 into LD915.	This study
LD4086	Δ*mpaR attB::*P*ccoN1-gfp*	Δ*mpaR* containing a construct in the *attB* site that expresses *gfp* under control of the 500 bp region upstream of *ccoN1*. Made by mating pLD2766 into LD3884.	This study
LD4087	Δ*mpaR attB::*P*ccoN2-gfp*	Δ*mpaR* containing a construct in the *attB* site that expresses *gfp* under control of the 500 bp region upstream of *ccoN2*. Made by mating pLD2767 into LD3884.	This study
LD4278	Δ*mpaR attB::*P*ccoN4-gfp*	Δ*mpaR* containing a construct in the *attB* site that expresses *gfp* under control of the 263 bp region upstream of *ccoN4*. Made by mating pLD4258 into LD3884.	This study
LD4344	Δ*hcn attB::*P*ccoN4-gfp*	Δ*hcnA* containing a construct in the *attB* site that expresses *gfp* under control of the 263 bp region upstream of *ccoN4*. Made by mating pLD4258 into LD2827.	This study
LD4895	Δ*hcn*Δ*mpaR* *attB::*P*ccoN4-gfp*	Δ*hcnA-C* and Δ*mpaR* containing a construct in the *attB* site that expresses *gfp* under control of the 263 bp region upstream of *ccoN4*. Made by mating pLD4258 into LD4867.	This study
LD4318	Δ*mpaR::mpaR* *attB::*P*ccoN4-gfp*	Δ*mpaR* containing a construct in the *attB* site that expresses *gfp* under control of the 263 bp region upstream of *ccoN4* with wild-type *mpaR* complemented back into the site of deletion. Made by mating pLD4286 into LD4277.	This study
LD4906	Δ*mpaR* attTn7::P*PA1/04/03-MpaR*	Δ*mpaR* containing a construct in the *attTn7* site that expresses the coding region of *mpaR* under control	This study

		of the *lac*-derived constitutive *PA1/04/03* promoter. Made by mating pLD4890 into LD3884.	
LD4907	Δ*mpaR* *attTn7::*P*PA1/04/03-MpaR* *attB::*P*ccoN4-gfp*	Δ*mpaR* containing a construct in the *attTn7* site that expresses the coding region of *mpaR* under control of the *lac*-derived constitutive *PA1/04/03* promoter and a construct in the *attB* site that expresses *gfp* under control of the 263 bp region upstream of *ccoN4*. Made by mating pLD4890 into LD4278.	This study
LD4908	Δ*hcn*Δ*mpaR* attTn7::P*PA1/04/03-MpaR*	Δ*hcnA-C* and Δ*mpaR* containing a construct in the *attTn7* site that expresses the coding region of *mpaR* under control of the *lac*-derived constitutive *PA1/04/03* promoter. Made by mating pLD4890 into LD4867.	This study
LD4909	Δ*hcn*Δ*mpaR* *attTn7::*P*PA1/04/03-MpaR* *attB::*P*ccoN4-gfp*	Δ*hcnA-C* and Δ*mpaR* containing a construct in the *attTn7* site that expresses the coding region of *mpaR* under control of the *lac*-derived constitutive *PA1/04/03* promoter and a construct in the *attB* site that expresses *gfp* under control of the 263 bp region upstream of *ccoN4*. Made by mating pLD4890 into LD4895.	This study
LD4281	PA14 *attB::*P*ccoN4(138bp)-gfp*	PA14 containing a construct in the *attB* site that expresses *gfp* under control of the 138 bp region upstream of *ccoN4*. Made by mating pLD4260 into PA14.	This study
LD4336	PA14 *attB::*P*ccoN4(129bp)-gfp*	PA14 containing a construct in the *attB* site that expresses *gfp* under control of the 129 bp region upstream of *ccoN4*. Made by mating pLD4310 into PA14.	This study
LD5000	PA14 *attB::*P*ccoN4(137bp shuffle)-gfp*	PA14 containing a construct in the *attB* site that expresses *gfp* under control of the 263 bp region upstream of *ccoN4* with 7 bp from 137-132 shuffled. Made by mating pLD4975 into PA14.	This study
LD4284	PA14 *attB::*P*ccoN4(147bp shuffle)-gfp*	PA14 containing a construct in the *attB* site that expresses *gfp* under control of the 263 bp region upstream of *ccoN4* with 10 bp from 147-138 shuffled. Made by mating pLD4263 into PA14.	This study
LD5001	PA14 attB::P*PA14_10540*-gfp	PA14 containing a construct in the *attB* site that expresses *gfp* under control of the ~400 bp surrounding the experimentally identified transcription start site of *PA14_10540*. Made by mating pLD4979 into PA14.	This study
LD4996	Δ*hcn* attB::P*PA14_10540*-gfp	Δ*hcnA-C* containing a construct in the *attB* site that expresses *gfp* under control of the ~400 bp surrounding the experimentally identified transcription start site of *PA14_10540*. Made by mating pLD4979 into LD2827.	This study

LD5037	Δ*mpaR* attB::P*PA14_10540*-gfp	PA14 containing a construct in the *attB* site that expresses *gfp* under control of the ~400 bp surrounding the experimentally identified transcription start site of *PA14_10540*. Made by mating pLD4979 into LD3884.	This study
LD5022	Δ*mpaR*::MpaR^{Y284A}	Y284 of *mpaR* (*PA14_10530*) mutated to alanine in the native locus. Made by mating pLD5006 into LD3884.	This study
LD5030	Δ*mpaR*::MpaR^{Y284A} attB::P*ccoN4-gfp*	Y284 of *mpaR* (*PA14_10530*) mutated to alanine in the native locus. Containing a construct in the *attB* site that expresses *gfp* under the control of the 263 bp region upstream of *ccoN4.* Made by mating pLD4258 into LD5022.	This study
LD5018	Δ*mpaR*::MpaR^{K314A}	K314 of *mpaR* (*PA14_10530*) mutated to alanine in the native locus. Made by mating pLD5007 into LD3884.	This study
LD5032	Δ*mpaR*::MpaR^{K314A} attB::P*ccoN4-gfp*	K314 of *mpaR* (*PA14_10530*) mutated to alanine in the native locus. Containing a construct in the *attB* site that expresses *gfp* under the control of the 263 bp region upstream of *ccoN4.* Made by mating pLD4258 into LD5018.	This study
LD4345	Δ*hcnB* attB::P*ccoN4-gfp*	Δ*hcnB* containing a construct in the *attB* site that expresses *gfp* under control of the 263 bp region upstream of *ccoN4*. Made by mating pLD4258 into LD2822.	This study
LD5160	Δ*hcnB::hcnB* attB::P*ccoN4-gfp*	PA14 Δ*hcnB* (*PA14_36320*) strain with wild-type *hcnB* complemented back into the site of deletion and a construct in the *attB* site that expresses *gfp* under the control of the 263 bp region upstream of *ccoN4*. Made by mating pLD5158 into LD4345.	This study
E. coli strains			
LD44	UQ950	E. coli DH5α λ(pir) host for cloning; F-Δ(*argF-lac*)169 Φ80 d*lacZ58*(ΔM15) *glnV44*(AS) *rfbD1 gyrA96*(NalR) *recA1 endA1 spoT1 thi-1 hsdR17 deoR* λpir+	D. Lies
LD661	BW29427	Donor strain for conjugation: *thrB1004 pro thi rpsL hsdS lacZ* ΔM15RP4–1360 Δ(*araBAD*)567 Δ*dapA1341*::[*erm pir*(wt)]	W. Metcalf
LD69	β2155	Helper strain. *thrB1004 pro thi strA hsdsS lacZ*ΔM15 (*F' lacZ*Δ*M15 lacI^q tra*Δ*36 proA$^+$ proB$^+$*) Δ*dapA::erm* (Erm^r)*pir::*RP4 [*::kan* (Km^r) from SM10]	(6)
LD2901	S17-1	Str^R, Tp^R, F$^-$ RP4-2-Tc::Mu *aphA*::Tn7 *recA* λpir lysogen	R. Simon

Saccharomyces cerevisiae strains			
LD676	InvSc1	*MATα/MATα leu2/leu2 trp1-289/trp1-289 ura3-52/ura3-52 his3-Δ1/his3-Δ1*	Invitro-gen

Table S2. Plasmids used in this study.

Plasmid Name	Description	Source
pMQ30	Yeast-based allelic-exchange vector; *sacB*$^+$, CEN/ARSH, URA3$^+$, Gm^R.	(7)
pFLP2	Site-specific excision vector with cl857-controlled FLP recombinase. encoding sequence, sacB$^+$, Amp^R. Used to insert LD2722-based plasmids into P. aeruginosa strains.	(8)
pLD2722	Gm^R, Tet^R flanked by Flp recombinase target (FRT) sites to resolve out resistance cassettes.	(4)
pAKN69	Gm^R, Cm^R mini-Tn7 $P_{PA1/04/03}$::*yfp*	(9)
pLD3774	Δ*mpaR* (*PA14_10530*) PCR fragment introduced into pMQ30 by gap repair cloning in yeast strain InvSc1.	This study
pLD4286	The CDS of *mpaR* with ~1 kb flanks on either side, introduced into pMQ30 by gap repair cloning in yeast strain InvSc1.	This study
pLD2766	500 bp upstream of *ccoN1* (*PA14_44340*) PCR fragment in pLD2722.	(4)
pLD2767	500 bp upstream of *ccoN2* (*PA14_44370*) PCR fragment in pLD2722.	(4)
pLD4258	263 bp upstream of *ccoN4* (*PA14_10500*) PCR fragment ligated into pLD2722 using SpeI and XhoI.	This study
pLD4260	138 bp upstream of *ccoN4* PCR fragment ligated into pLD2722 using SpeI and XhoI.	This study
pLD4310	129 bp upstream of *ccoN4* PCR fragment ligated into pLD2722 using SpeI and XhoI.	This study
pLD4975	263 bp upstream of *ccoN4* with the 7 bp between 137 and 132 before the ATG shuffled PCR fragment ligated into pLD2722 using SpeI and XhoI.	This study
pLD4263	263 bp upstream of *ccoN4* with the 10 bp between 147 and 138 before the ATG shuffled PCR fragment ligated into pLD2722 using SpeI and XhoI.	This study
pLD4890	The CDS of *mpaR* with lambda t0 terminator PCR fragment ligated into pAKN69 using NheI and SphI.	This study

pLD4979	396 bp upstream to 30 bp downstream of PA14 genomic locus: 908280 PCR fragment ligated into pLD2722 using SpeI and XhoI.	This study
pLD5006	The CDS of *mpaR* with ~1 kb flanks on either side with Y284 changed to an alanine, introduced into pMQ30 by gap repair cloning in yeast strain InvSc-1	This study
pLD5007	The CDS of *mpaR* with ~1 kb flanks on either side with K314 changed to an alanine, introduced into pMQ30 by gap repair cloning in yeast strain InvSc-1	This study
pLD2790	Δ*hcnB* (*PA14_36320*) PCR fragment introduced into pMQ30 by gap repair cloning in yeast strain InvSc1.	This study
pLD5158	The CDS of *hcnB* with ~1 kb flanks on either side, introduced into pMQ30 by gap repair cloning in yeast strain InvSc1.	This study

Table S3. Primers used in this study.

Primer Number	Sequence
Primers for plasmid pLD3774 (used to make Δ*mpaR*)	
3236	caggcaaattctgttttatcagaccgcttctgcgttctGACCTGGAAAGCCTGTTCTG
3237	gtctccatcgccttctcctgGTAGGTACGACTGGCGTGC
3238	ggcacgccagtcgtacctacCAGGAGAAGGCGATGGAG
3239	ggaattgtgagcggataacaatttcacacaggaaacagctGCCCAGATGTACAGGGTGAT
Primers for plasmid pLD4286 (used to make *mpaR* complement)	
3797	caggcaaattctgttttatcagaccgcttctgcgttctGACAAGGACACCCTGATCGT
3798	CAACTCCAGCCAGAGGAAGT
3799	GCAGTTCCTTCTCCAAGAGC
3800	ggaattgtgagcggataacaatttcacacaggaaacagctAGGTGGACATGCCGTAGAAC
Primers for plasmid pLD4258 (used to make P*ccoN4-gfp*)	
3855	gattcgactgcactagtCGCCCCGCACACG
3701	gattcgactgcctcgagCTGTACAGTCCCGAAAGAAATGA
Primers for plasmid pLD4260 (used to make P*ccoN4(138bp)-gfp*)	
3860	gattcgactgcactagtCATCTGATACCCAAATTCCATATC
3701	gattcgactgcctcgagCTGTACAGTCCCGAAAGAAATGA

Primers for plasmid pLD4309 (used to make P*ccoN4(129bp)-gfp*)	
3900	gattcgactgcactagtCCCAAATTCCATATCGACCTG
3701	gattcgactgcctcgagCTGTACAGTCCCGAAAGAAATGA
Primers for plasmid pLD4975 (used to make P*ccoN4(137bp shuffle)-gfp*)	
3855	gattcgactgcactagtCGCCCCGCACACG
4491	CTTCCCCCGCCCCGATATCAACCCAAATTCCATATCGACCT
4492	AGGTCGATATGGAATTTGGGTTGATATCGGGGCGGGGGAAG
3701	gattcgactgcctcgagCTGTACAGTCCCGAAAGAAATGA
Primers for plasmid pLD4263 (used to make P*ccoN4(147bp shuffle)-gfp*)	
3855	gattcgactgcactagtCGCCCCGCACACG
3857	CGATATGGAATTTGGGTATCAGATatatatatatAAGGCGTTCGTGCAGG
3858	CCTGCACGAACGCCTTatatatatatATCTGATACCCAAATTCCATATCG
3701	gattcgactgcctcgagCTGTACAGTCCCGAAAGAAATGA
Primers for plasmid pLD4890 (used to make P*PA1/04/03-mpaR*)	
4387	gattcgactgcgcatgctgAAGCGCTACGAGAAATTCG
4388	actggatctatcaacaggagtccaaTCATCCCGCCAGCG
4389	GATCGCCCAGTCGCTGGCGGGATGAttggactcctgttgatagatccag
4077	acgtacgtacgctagcTTGGATTCTCACCAATAAAAAACGCC
Primers for plasmid pLD4979 (used to make P*PA14_10540-gfp*)	
4496	gattcgactgcactagtGCCTGGTCGTATTCGTCGTA
4497	gattcgactgcctcgagTTTTCGTAGGCCCATCAGG
Primers for plasmid pLD5006 (used to make *mpaR*^{Y284A})	
4502	caggcaaattctgttttatcagaccgcttctgcgttctCGATCACCTTCATCGGCTA
4503	gccgaagtacagttcggcggcCACGTCGTCCTCGATCATC
4504	gatgatcgaggacgacgtggccGCCGAACTGTACTTCGGC
4505	ggaattgtgagcggataacaatttcacacaggaaacagctGCCCAGATGTACAGGGTGAT
Primers for plasmid pLD5007 (used to make *mpaR*^{K314A})	
4502	caggcaaattctgttttatcagaccgcttctgcgttctCGATCACCTTCATCGGCTA

4506	gtagcccggcgccaggctcgcGGAGAAGGAACTGCAGTGC
4507	gtgatgcactgcagttccttctccgcgAGCCTGGCGCCGG
4505	ggaattgtgagcggataacaatttcacacaggaaacagctGCCCAGATGTACAGGGTGAT
Primers for plasmid pLD2790 (used to make ∆*hcnB*)	
2160	ccaggcaaattctgttttatcagaccgcttctgcgttctgatGTTGACCTGTTGCAGCTCC
2161	gtcccctgcatcctcttcgTCGATCCGATCCCGTTTTCC
2162	ggaaaacgggatcggatcgaCGAAGAGGATGCAGGGGAC
2163	ggaattgtgagcggataacaatttcacacaggaaacagctGCTGGTCGACACAGATGAAC
Primers for plasmid pLD5158 (used to make *hcnB* complement)	
2160	ccaggcaaattctgttttatcagaccgcttctgcgttctgatGTTGACCTGTTGCAGCTCC
2163	ggaattgtgagcggataacaatttcacacaggaaacagctGCTGGTCGACACAGATGAAC

SUPPLEMENTAL REFERENCES

1. Wang T, Qi Y, Wang Z, Zhao J, Ji L, Li J, Cai Z, Yang L, Wu M, Liang H. 2020. Coordinated regulation of anthranilate metabolism and bacterial virulence by the GntR family regulator MpaR in Pseudomonas aeruginosa. Mol Microbiol 114:857–869.

2. Frangipani E, Pérez-Martínez I, Williams HD, Cherbuin G, Haas D. 2014. A novel cyanide-inducible gene cluster helps protect Pseudomonas aeruginosa from cyanide. Environ Microbiol Rep 6:28–34.

3. Rahme LG, Stevens EJ, Wolfort SF, Shao J, Tompkins RG, Ausubel FM. 1995. Common virulence factors for bacterial pathogenicity in plants and animals. Science 268:1899–1902.

4. Jo J, Cortez KL, Cornell WC, Price-Whelan A, Dietrich LE. 2017. An orphan cbb3-type cytochrome oxidase subunit supports Pseudomonas aeruginosa biofilm growth and virulence. Elife 6.

5. Lin Y-C, Sekedat MD, Cornell WC, Silva GM, Okegbe C, Price-Whelan A, Vogel C, Dietrich LEP. 2018. Phenazines regulate Nap-dependent denitrification in Pseudomonas aeruginosa biofilms. J Bacteriol https://doi.org/10.1128/JB.00031-18.

6. Dehio C, Meyer M. 1997. Maintenance of broad-host-range incompatibility group P and group Q plasmids and transposition of Tn5 in Bartonella henselae following conjugal plasmid transfer from Escherichia coli. J Bacteriol 179:538–540.

7. Shanks RMQ, Caiazza NC, Hinsa SM, Toutain CM, O'Toole GA. 2006. Saccharomyces cerevisiae-based molecular tool kit for manipulation of genes from gram-negative bacteria. Appl Environ Microbiol 72:5027–5036.

8. Hoang TT, Karkhoff-Schweizer RR, Kutchma AJ, Schweizer HP. 1998. A broad-host-range Flp-FRT recombination system for site-specific excision of chromosomally-located DNA sequences: application for isolation of unmarked Pseudomonas aeruginosa mutants. Gene 212:77–86.

9. Lambertsen L, Sternberg C, Molin S. 2004. Mini-Tn7 transposons for site-specific tagging of bacteria with fluorescent proteins. Environ Microbiol 6:726–732.
